# Structural basis of the strict specificity of a bacterial GH31 α-1,3-glucosidase for nigerooligosaccharides

**DOI:** 10.1016/j.jbc.2022.101827

**Published:** 2022-03-12

**Authors:** Marina Ikegaya, Toshio Moriya, Naruhiko Adachi, Masato Kawasaki, Enoch Y. Park, Takatsugu Miyazaki

**Affiliations:** 1 Department of Bioscience, Graduate School of Science and Technology, Shizuoka University, Shizuoka, Japan; 2 Structural Biology Research Center, Institute of Materials Structure Science, High Energy Accelerator Research Organization (KEK), Tsukuba, Ibaraki, Japan; 3 Department of Materials Structure Science, School of High Energy Accelerator Science, The Graduate University of Advanced Studies (Soken-dai), Tsukuba, Ibaraki, Japan; 4 Research Institute of Green Science and Technology, Shizuoka University, Shizuoka, Japan

**Keywords:** α-1,3-glucosidase, carbohydrate metabolism, cryogenic electron microscopy, enzyme mechanism, glycoside hydrolase, glycoside hydrolase family 31, hexamer, nigerose, oligosaccharide, X-ray crystallography, CADE, GH31 cycloalternan-specific α-1,3-glucosidase, cryo-EM, cryogenic electron microscopy, CTF, contrast transfer function, CtGII, α-glucosidase II from *Chaetomium thermophilum* var. *thermophilum*, ER, endoplasmic reticulum, GHs, glycoside hydrolases, MGAM, mammalian intestinal maltase-glucoamylase, NtMGAM, N-terminal maltase domain of human maltase-glucoamylase, PDB, Protein Data Bank, pNP-α-Glc, *p*-nitrophenyl α-D-glucopyranoside

## Abstract

Carbohydrate-active enzymes are involved in the degradation, biosynthesis, and modification of carbohydrates and vary with the diversity of carbohydrates. The glycoside hydrolase (GH) family 31 is one of the most diverse families of carbohydrate-active enzymes, containing various enzymes that act on α-glycosides. However, the function of some GH31 groups remains unknown, as their enzymatic activity is difficult to estimate due to the low amino acid sequence similarity between characterized and uncharacterized members. Here, we performed a phylogenetic analysis and discovered a protein cluster (GH31_u1) sharing low sequence similarity with the reported GH31 enzymes. Within this cluster, we showed that a GH31_u1 protein from *Lactococcus lactis* (LlGH31_u1) and its fungal homolog demonstrated hydrolytic activities against nigerose [α-D-Glc*p*-(1→3)-D-Glc]. The *k*_cat_/*K*_m_ values of LlGH31_u1 against kojibiose and maltose were 13% and 2.1% of that against nigerose, indicating that LlGH31_u1 has a higher specificity to the α-1,3 linkage of nigerose than other characterized GH31 enzymes, including eukaryotic enzymes. Furthermore, the three-dimensional structures of LlGH31_u1 determined using X-ray crystallography and cryogenic electron microscopy revealed that LlGH31_u1 forms a hexamer and has a C-terminal domain comprising four α-helices, suggesting that it contributes to hexamerization. Finally, crystal structures in complex with nigerooligosaccharides and kojibiose along with mutational analysis revealed the active site residues involved in substrate recognition in this enzyme. This study reports the first structure of a bacterial GH31 α-1,3-glucosidase and provides new insight into the substrate specificity of GH31 enzymes and the physiological functions of bacterial and fungal GH31_u1 members.

Carbohydrates are widely distributed in nature, and their structures are diverse as per their physiological functions. The biosynthesis and degradation of carbohydrates are catalyzed by carbohydrate-active enzymes, including glycoside hydrolases (GHs), glycosyltransferases, and polysaccharide lyases, and are classified into many families based on sequence similarity in the CAZy database (http://www.cazy.org/) (1, 2). GHs are involved in carbohydrate degradation and glycoside formation through transglycosylation reactions (3, 4). There are >160 GH families, and recent studies have discovered new enzymes in various microorganisms (5, 6). However, there are many putative GHs with unknown functions among the known GH families.

α-1,4-Glucosidases (EC 3.2.1.20) hydrolyze α-1,4-glucosidic linkages at the nonreducing end of substrates and release α-glucose *via* the anomer-retaining reaction mechanism. The enzymes are present in numerous organisms and primarily belong to the GH13 and GH31 families (7). GH31 is one of the most diversified GH families and harbors not only α-1,4-glucosidases but also α-1,3-glucosidases (8), endoplasmic reticulum (ER) α-glucosidase II (9, 10), α-xylosidases (11), α-galactosidases (12), sulfoquinovosidases (13), α-*N*-acetylgalactosaminidases (14, 15, 16), and α-glucan lyases (17). GH31 α-glucoside hydrolases also vary in their function and substrate specificity; these include mammalian intestinal maltase-glucoamylase (MGAM) (18, 19), sucrase-isomaltase (20), and lysosomal acid α-glucosidase, which catalyzes glycogen breakdown (21). Some GH31 enzymes can efficiently catalyze transglycosylation and produce oligosaccharides containing α-1,3- and α-1,6-glucosidic bonds (22, 23). Furthermore, some bacterial GH31 enzymes catalyze unique reactions. While most known GH31 enzymes are *exo*-acting, a GH31 dextranase derived from *Flavobacterium johnsoniae* (FjDex31A) is *endo*-acting and releases isomaltooligosaccharides from dextran (24, 25). An α-transglucosylase derived from *Cellvibrio japonicus* (Agd31B) transfers single glucosyl units from α-1,4-glucans to the 4-OH group of glucose (26). Cycloalternan [cyclic α-nigerosyl-(1→6)-nigerose] is formed by two GH31 transglucosidases, namely, 6-α-glucosyltransferase and cycloalternan-forming enzyme (3-α-isomaltosyltransferase) and degraded by GH31 cycloalternan-specific α-1,3-glucosidase (CADE) (3). Despite differences in their substrate specificities and reactions, the characterized GH31 enzymes share two aspartic acid residues as catalytic residues in the anomer-retaining catalytic mechanism (27).

Although many GH31 enzymes have been characterized to date, there are still many proteins in microbial genomes whose activities are unclear. The diversity of the GH31 family indicates the existence of novel enzymes. Phylogenetic analysis and the subclassification of the enzyme family is a powerful strategy for discovering new enzymes from a large sequence space (27, 28). Recently, some open-source software and servers for protein sequence analysis have been developed for handling a large dataset (28, 29, 30). In the present study, we performed a phylogenetic analysis of GH31 proteins and identified new microbial members of the GH31 family, which showed strict α-1,3-glucosidase activities on nigerooligosaccharides. Furthermore, using X-ray crystallography and cryogenic electron microscopy (cryo-EM), the three-dimensional structure of the bacterial members was determined to discuss their substrate recognition mechanism.

## Results and discussion

### GH31 uncharacterized protein cluster

**Figure 1:**
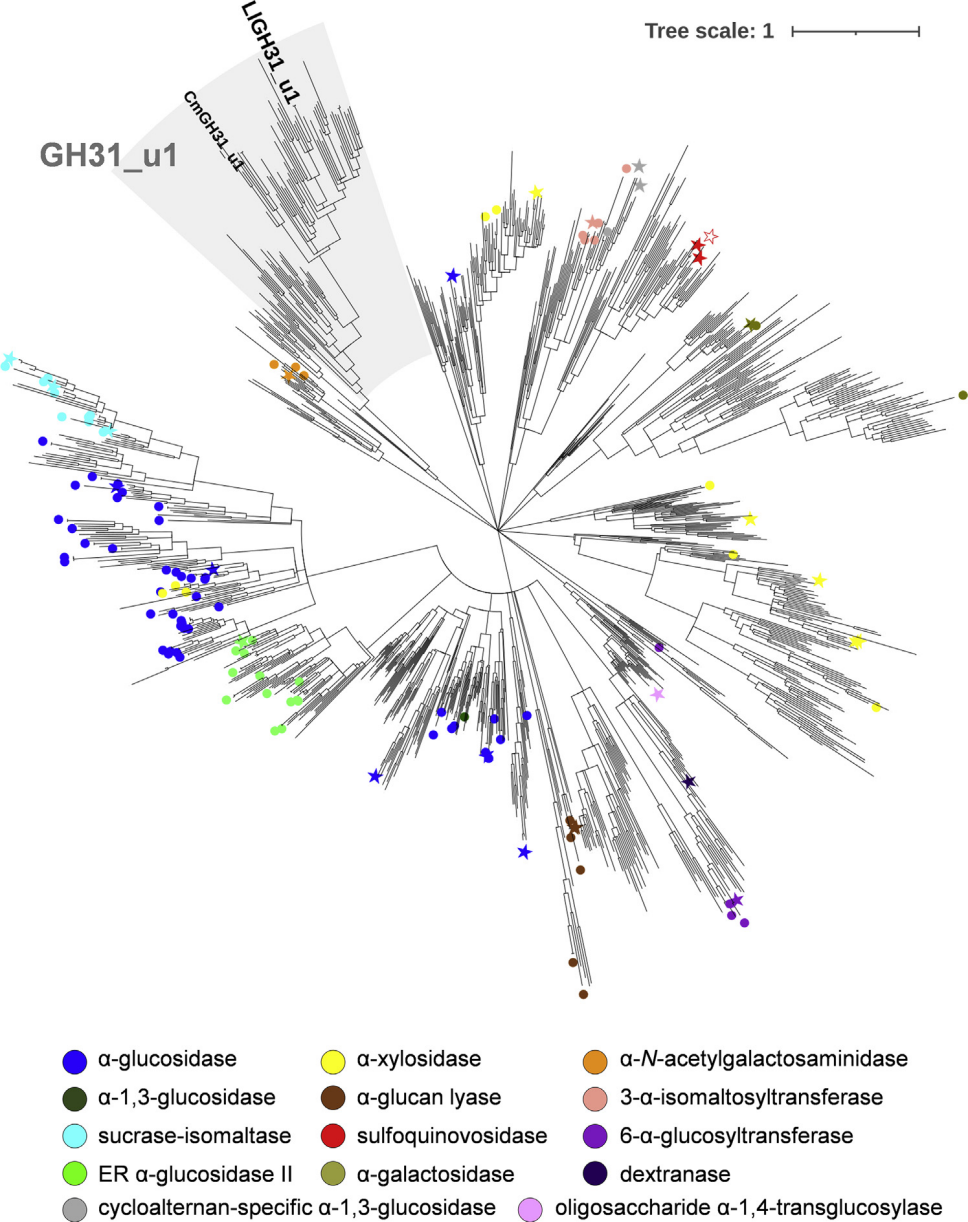
**Phylogenetic tree of GH31 proteins.** A total of 1194 GH31 sequences were used to generate the phylogenetic tree. GH31 catalytic domains were aligned using the MUSCLE algorithm, and the phylogenetic tree was built using the maximum likelihood method with MEGA X (53). Symbols: *solid circle*, the enzymatically characterized proteins; *solid star*, the enzymatically characterized and structure-determined proteins; *open star*, the protein whose structure has been deposited on PDB. Symbol colors are listed below the tree. The clade of GH31_u1 proteins is highlighted with gray background. GH, glycoside hydrolase.

We performed a phylogenetic analysis using protein sequences classified as the GH31 family on the CAZy database. Protein sequences sharing <70% identity extracted from all GH31 sequences and characterized sequences (1194 sequences) were subjected to the phylogenetic analysis (Fig. 1). A clade of uncharacterized proteins derived from bacteria and fungi was detected (Fig. S1). These proteins are classified as GH31_u1 family in National Center for Biotechnology Information Conserved Domain Database and showed <25% amino acid sequence identity with the previously characterized GH31 enzymes, indicating that the activities and substrate specificities of GH31_u1 proteins differed from those of characterized GH31 enzymes. In this study, GH31_u1 proteins from *Lactococcus lactis* subsp. *cremoris* MG1363 (named LlGH31_u1) and *Cordyceps militaris* (named CmGH31_u1) were targeted to clarify the functions and structures of GH31_u1 proteins. LlGH31_u1 is the sole GH31 protein encoded in the genome of *L. lactis* subsp. *cremoris*, whereas the *C. militaris* has three other GH31 proteins (not GH31_u1) compared with CmGH31_u1. LlGH31_u1 and CmGH31_u1 share 37.5% amino acid sequence identity, have no signal peptide, and have no domain annotated in the Conserved Domains Database in addition to the conserved GH31 region, indicating that they possibly function intracellularly.

### Enzymatic features of recombinant GH31_u1 proteins

N-terminally His-tagged LlGH31_u1 and CmGH31_u1 were expressed in *Escherichia coli* and purified to homogeneity. The yields of the purified LlGH31_u1 and CmGH31_u1 were 2 and 0.1 mg from 1 L of culture, respectively. These enzymes were active against only *p*-nitrophenyl α-D-glucopyranoside (pNP-α-Glc) among the *p*-nitrophenyl glycosides tested, suggesting that they are α-glucoside hydrolases. The effects of pH and temperature on the hydrolytic activity of LlGH31_u1 were evaluated using pNP-α-Glc as a substrate. LlGH31_u1 showed the highest activity at pH 7.0 for 5-min incubation at 30 °C and maintained >79% of its maximal activity in the pH range of 4.5 to 8.0 after a 20-h incubation at 4 °C. The enzyme showed the highest activity at 35 °C for 5-min incubation at pH 7.0, but it was unstable at temperatures >35 °C (<63%) and approximately 5% of its activity remained after a 30-min incubation at 40 °C (Fig. S2). CmGH31_u1 exhibited the highest activity at pH 6.0 and 40 °C for 5-min incubation in the condition described in Experimental procedures (Fig. S3).

**Figure 2:**
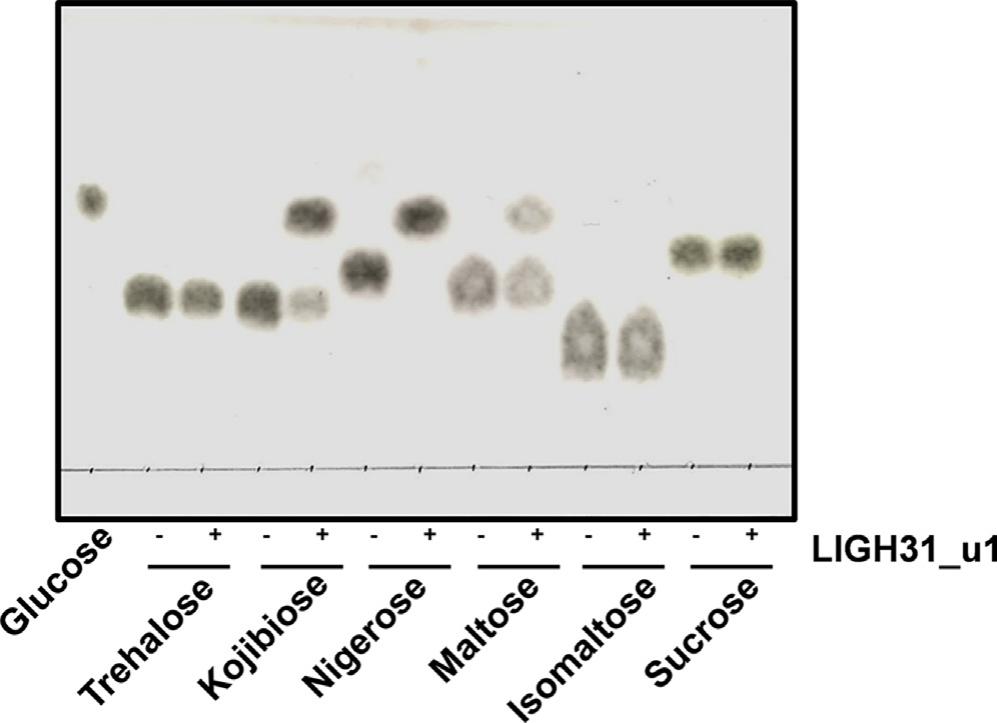
**TLC analysis of LlGH31_u1 hydrolysis toward disaccharides.** LlGH31_u1 (0.1 mg/ml) was incubated with 10 mM disaccharides (trehalose, kojibiose, nigerose, maltose, isomaltose, and sucrose) for 1 h at 30 °C. Glucose standard and the reaction mixtures were developed on a TLC plate with 1-butanol/ethanol/water (10:5:2, vol/vol).

Next, we analyzed hydrolytic activities toward disaccharides, trehalose [α-D-Glc*p*-(1↔1)-α-D-Glc*p*], kojibiose [α-D-Glc*p*-(1→2)-D-Glc], nigerose [α-D-Glc*p*-(1→3)-D-Glc], maltose [α-D-Glc*p*-(1→4)-D-Glc], isomaltose [α-D-Glc*p*-(1→6)-D-Glc], and sucrose [β-D-Fru*f*-(2↔1)-α-D-Glc*p*] using thin-layer chromatography (TLC). After a 1-h incubation with 0.1 mg/ml LlGH31_u1, only nigerose was completely hydrolyzed; the hydrolysis of kojibiose and maltose was also observed (Fig. 2). Both LlGH31_u1 and CmGH31_u1 exhibited the highest hydrolytic activity toward nigerose, followed by kojibiose, maltose, and isomaltose in that order (Table 1). We detected no hydrolytic activity when either trehalose or sucrose was used as a substrate. The *K*_m_ and *k*_cat_ values of nigerose were 9.2 ± 0.6 mM and 9.7 ± 0.3 s^−1^, respectively (Table 2). By contrast, the *K*_m_ value of kojibiose was almost the same as that of nigerose, and the *k*_cat_ value was 8.6 times lower than that of nigerose. The higher *K*_m_ and lower *k*_cat_ values were observed when maltose was employed as a substrate. We then analyzed the substrate specificity of LlGH31_u1 for longer oligosaccharides. The *K*_m_ values of nigerotetraose and nigerotriose were approximately three times lower than that of nigerose. The *K*_m_ values of LlGH31_u1 for nigerose are in the same range (millimolar order) as those of other GH31 α-glucosidases for their preferred substrates (Table S1). However, maltotriose and maltotetraose have higher *K*_m_ values than nigerose. LlGH31_u1 did not release glucose from nigeran, an insoluble linear α-glucan with alternating α-1,3- and α-1,4-glucosidic linkages derived from *Aspergillus niger*.

**Table 1:** Activities of LlGH31_u1 WT, Y99F, and CmGH31_u1 against disaccharides

Substrate	LlGH31_u1 WT		LlGH31_u1 Y99F		CmGH31_u1	
	Activity (μmol/mg/min)	Relative activity^a^ (%)	Activity (μmol/mg/min)	Relative activity (%)	Activity (μmol/mg/min)	Relative activity (%)
Trehalose	ND^b^	ND	ND	ND	ND	ND
Kojibiose	0.34 ± 0.02	5.2	(49 ± 0.7) × 10^−3^	1.3	(2.4 ± 0.1) × 10^−2^	4.2
Nigerose	6.3 ± 0.5	100	3.9 ± 0.2	100	(5.6 ± 0.7) × 10^−2^	100
Maltose	(10 ± 0.8) × 10^−3^	0.16	(20 ± 11) × 10^−3^	0.53	(8.2 ± 3.8) × 10^−3^	1.5
Isomaltose	(2.9 ± 3.5) × 10^−3^	0.045	(5.4 ± 0.2) × 10^−3^	0.15	(8.4 ± 0.3) × 10^−3^	1.5
Sucrose	ND	ND	ND	ND	ND	ND

a Each activity for nigerose is taken as 100%.

b ND, not detected.

**Table 2:** Kinetic parameters of LlGH31_u1 and CmGH31_u1

Enzyme	Substrate	*K*_m_ (mM)	*k*_cat_ (s ^−1^)	*k*_cat_/*K*_m_ (s^−1^ mM^−1^)	Relative *k*_cat_/*K*_m_ (%)^a^
LlGH31_u1 WT	pNP-α-Glc	20 ± 0.8	31 ± 0.7	1.5 ± 0.08	145
	Nigerose	9.2 ± 0.6	9.7 ± 0.3	1.1 ± 0.2	100
	Nigerotriose	3.1 ± 0.4	8.1 ± 0.4	2.6 ± 0.3	246
	Nigerotetraose	2.9 ± 0.2	5.6 ± 0.2	1.9 ± 0.2	182
	Kojibiose	8.2 ± 0.4	1.1 ± 0.02	(1.4 ± 0.05) × 10^−1^	13
	Maltose	36 ± 1.9	(7.9 ± 0.2) × 10^−1^	(2.2 ± 0.03) × 10^−2^	2.1
	Maltotriose	39 ± 2.7	(6.2 ± 0.2) × 10^−1^	(1.6 ± 0.09) × 10^−2^	1.5
	Maltotetraose	82 ± 17	(1.5 ± 0.2) × 10^−1^	(1.8 ± 0.4) × 10^−3^	0.2
LlGH31_u1 Y99F	pNP-α-Glc	28 ± 0.8	59 ± 0.8	2.1 ± 0.02	204
	Nigerose	12 ± 2.0	5.2 ± 0.4	(4.3 ± 0.2) × 10^−1^	38
	Nigerotriose	2.0 ± 0.2	4.9 ± 0.4	2.3 ± 0.9	221
CmGH31_u1	pNP-α-Glc	4.7 ± 0.8	4.1 ± 0.3	(8.9 ± 2) × 10^−1^	
	Nigerose	6.3 ± 0.6	(4.5 ± 0.3) × 10^−1^	(7.2 ± 1) × 10^−2^	
	Nigerotriose	2.0 ± 0.6	(4.7 ± 0.3) × 10^−1^	(2.3 ± 0.4) × 10^−1^	

a *k*_cat_/*K*_m_ value of LlGH31_u1 WT for nigerose is taken as 100%.

Based on the CAZy classification, while α-1,4-glucosidases (EC 3.2.1.20) are present in the GH families 4, 13, 31, and 122 (1, 27, 31, 32), the GH31 and GH63 families contain nigerose-active α-glucoside hydrolases (7, 33). However, YgjK, a GH63 enzyme, from *E. coli* had a high *K*_m_ value (approximately 230 mM) for nigerose (33), and its natural substrate was assumed to be another α-glucoside (34, 35). Other than GHs, a nigerose phosphorylase from *Lachnoclostridium phytofermentans* (formerly *Clostridium phytofermentans*) (Cphy1874) belonging to the GH65 family displays high specificity against nigerose (Table S1), but it does not catalyze hydrolysis (36). At present, the catalytic α subunit of ER α-glucosidase II, CADE (3, 4), and *Lactobacillus johnsonii* α-1,3-glucosidase (LJAG31) (8) have been identified as GH31 enzymes that display specificity against α-1,3-glucosidic linkage. ER α-glucosidase II hydrolyzes α-1,3 linkages in the α-Glc-(1→3)-Glc and α-Glc-(1→3)-mannose moieties of *N*-glycans. Despite its biological role in cleaving α-1,3-linked glucose, ER α-glucosidase II has a significant *k*_cat_/*K*_m_ value on maltose (27%–50% of that for nigerose, Table S1) (37). As shown in Figure 1, mammalian α-glucosidases, *i.e.*, ER α-glucosidase II, MGAM, sucrase-isomaltase, and lysosomal acid α-glucosidase, are phylogenetically close, and this agrees with the enzymatic feature acting on both the α-1,3 and α-1,4 bonds. LJAG31 displays the highest nigerose hydrolytic activity, although it exhibits a broad substrate specificity; the *k*_cat_/*K*_m_ values for maltulose [α-D-Glc*p*-(1→4)-D-Fru] and kojibiose were 73% and 61%, respectively, of those for nigerose (8). CADE is cycloalternan-specific, and its hydrolytic activity against nigerose is <0.1% of that for cycloalternan (4). However, the *k*_cat_/*K*_m_ value of LlGH31_u1 against other glucobiose is <13% of that against nigerose, and this enzyme exhibits the highest substrate specificity for nigerose among the characterized GH31 enzymes.

### Crystal structure of LlGH31_u1

**Figure 3:**
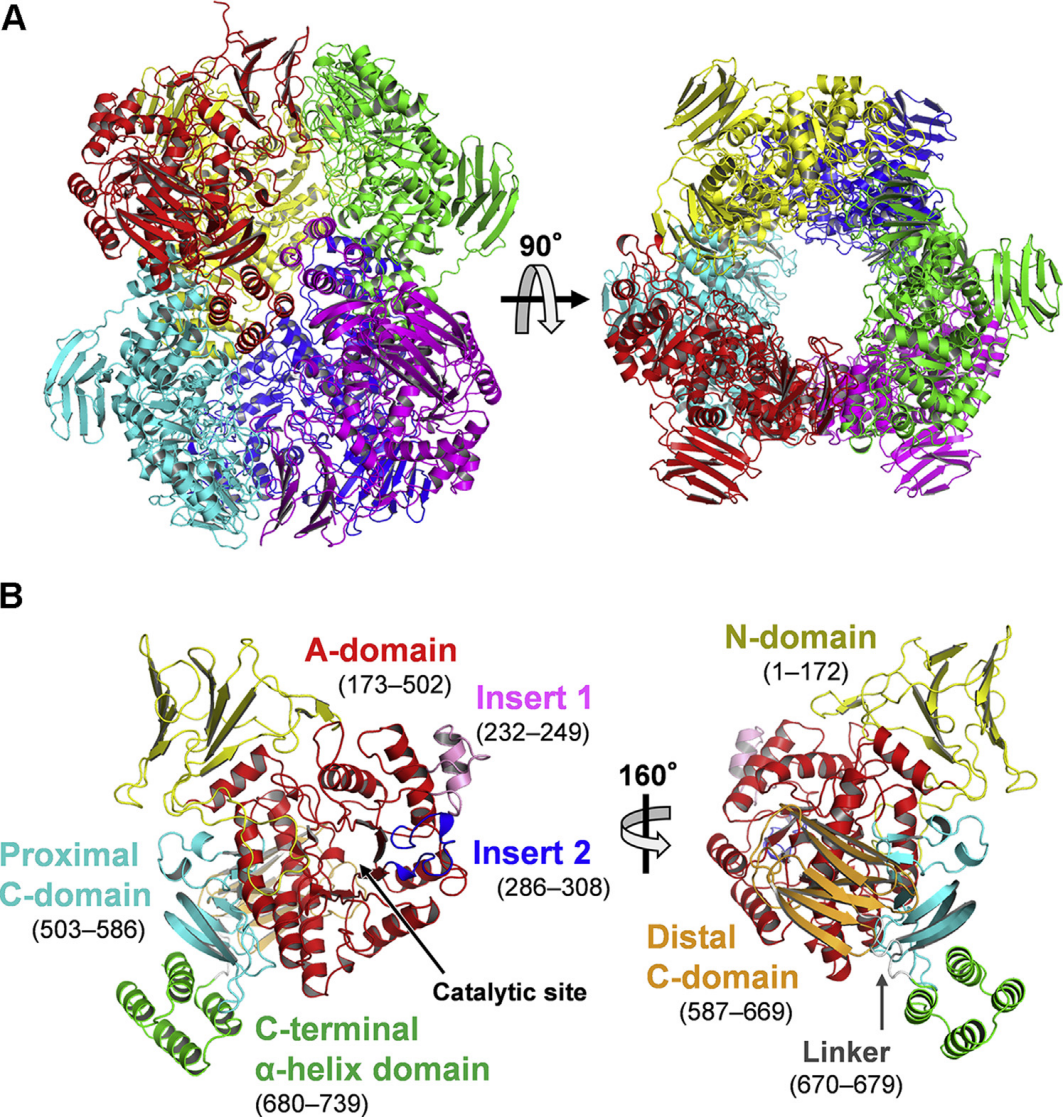
**Overall structure of LlGH31_u1 determined using X-ray crystallography.***A*, hexameric structure of LlGH31_u1 determined using the crystal form 1 in which the space group is *P*2_1_. Each monomer is shown in different colors. *B*, ribbon model of LlGH31_u1 monomer. *Left*, view from the front of the active site cleft; *right*, view rotated to adjust to the same orientation as the monomer colored in *red* in the *right panel* of (*A*). Individual domains are colored as follows: N-domain, *yellow*; A-domain, *red*; insert 1, *pink*; insert 2, *blue*; proximal C-domain, *cyan*; distal C-domain, *orange*; linker, *gray*; and C-terminal α-helix domain, *green*.

WT LlGH31_u1 was crystallized in two different crystal forms (named, crystal forms 1 and 2, see Experimental procedures). Crystal forms 1 and 2 belong to the space groups *P*2_1_ and *P*6_3_22, respectively, and comprise six and one molecule of LlGH31_u1, respectively, in an asymmetric unit. The *P*2_1_ and *P*6_3_22 structures of unliganded WT (named WT*_P*2_1_ and WT_*P*6_3_22, respectively) were determined at 1.75- and 1.85-Å resolutions (Fig. 3*A* and Table S2). Despite the space group difference, hexamer formation could be generated *via* the symmetry operations in the *P*6_3_22 space group, and the hexamer assembly is isomorphous with six monomers in WT*_P*2_1_. LlGH31_u1 has a theoretical mass of 85.7 kDa and a molecular weight of 453 kDa as determined using gel filtration chromatography. With molecular assembly in the crystal structures, LlGH31_u1 probably forms a hexamer. Two GH31 enzymes, α-xylosidase YicI from *E. coli* and α-glucosidase MalA from *Sulfolobus solfataricus*, reportedly formed a hexamer (11, 38); however, the hexameric arrangement of LlGH31_u1 differs from theirs (Fig. S4).

The monomeric structure of LlGH31_u1 contains four domains that are generally conserved in GH31 enzymes: N-terminal β-sandwich domain (N-domain, residues 1–172); (β/α)_8_-barrel catalytic domain (A-domain, residues 173–502) with two inserted components insert 1 (residues 232–249) and insert 2 (residues 286–308) located between β3 and α4 and between β4 and α5, respectively; proximal C-domain (residues 503–586); and distal C-domain (residues 587–669). An extra domain consisted of four α-helices (residues 680–739) at the C terminus connected by a linker polypeptide (residues 670–679) (Fig. 3*B*). Interactions between insertions in the A-domain and a β-sheet of the N-domain from an adjacent protomer as well as the C-terminal α-helix domain contribute to hexamer formation (Figs. 3*A* and S5). We performed a structural similarity search on the DALI server (39) using WT_*P*6_3_22 as a query. An α-xylosidase from *Bacteroides ovatus* [BoGH31; Protein Data Bank (PDB) 5JOV; Z = 33.1; amino acid sequence identity = 23%] had the highest Z-score, followed by a cycloalternan-degrading enzyme from *Trueperella pyogenes* (TpCADE; PDB 5I0G; Z = 32.1; identity = 19%) and other structurally identified GH31 enzymes. The DALI results also included ER α-glucosidase II from *Chaetomium thermophilum* var. *thermophilum* (CtGII, 5DKY, Z = 28.1; identity = 19%), similarly as other GH31 enzymes. A DALI search using the C-terminal α-helix domain revealed that it has the highest structural similarity with harmonin homology domain 2 of whirlin (PDB 6FDD, Z = 5.8), which is expressed in hair and photoreceptor cells and is essential for sound and light perception, although their amino acid sequence identity was 12%. The harmonin homology domain of cerebral cavernous malformations 2 (PDB 4FQN) was reported to be involved in dimer formation (40). Although these proteins and LlGH31_u1 have no physiological function in common, this α-helix fold may be relevant in oligomer formation.

### Comparison of cryo-EM and crystal structures of LlGH31_u1

**Figure 4:**
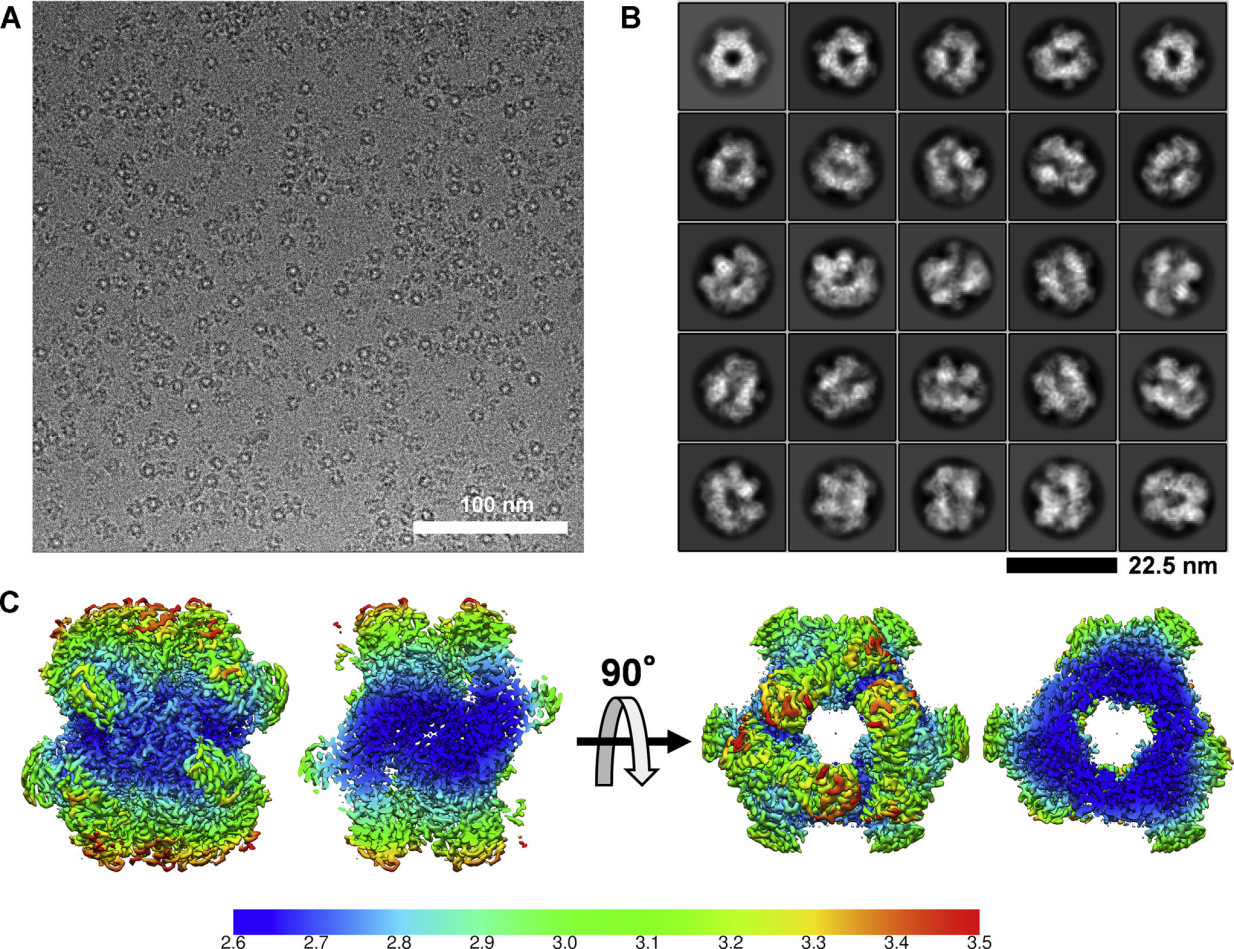
**Cryo-EM single-particle analysis of LlGH31_u1.***A and B*, representative micrograph (*A*) and two-dimensional class averages of the cryo-EM dataset (*B*). *C*, local resolution of final cryo-EM structure. Unit of color bar label is in Å. The diagram is viewed from the same orientation as the ribbon models in Figures 3*A* and 5*A*. cryo-EM, cryogenic electron microscopy.

**Figure 5:**
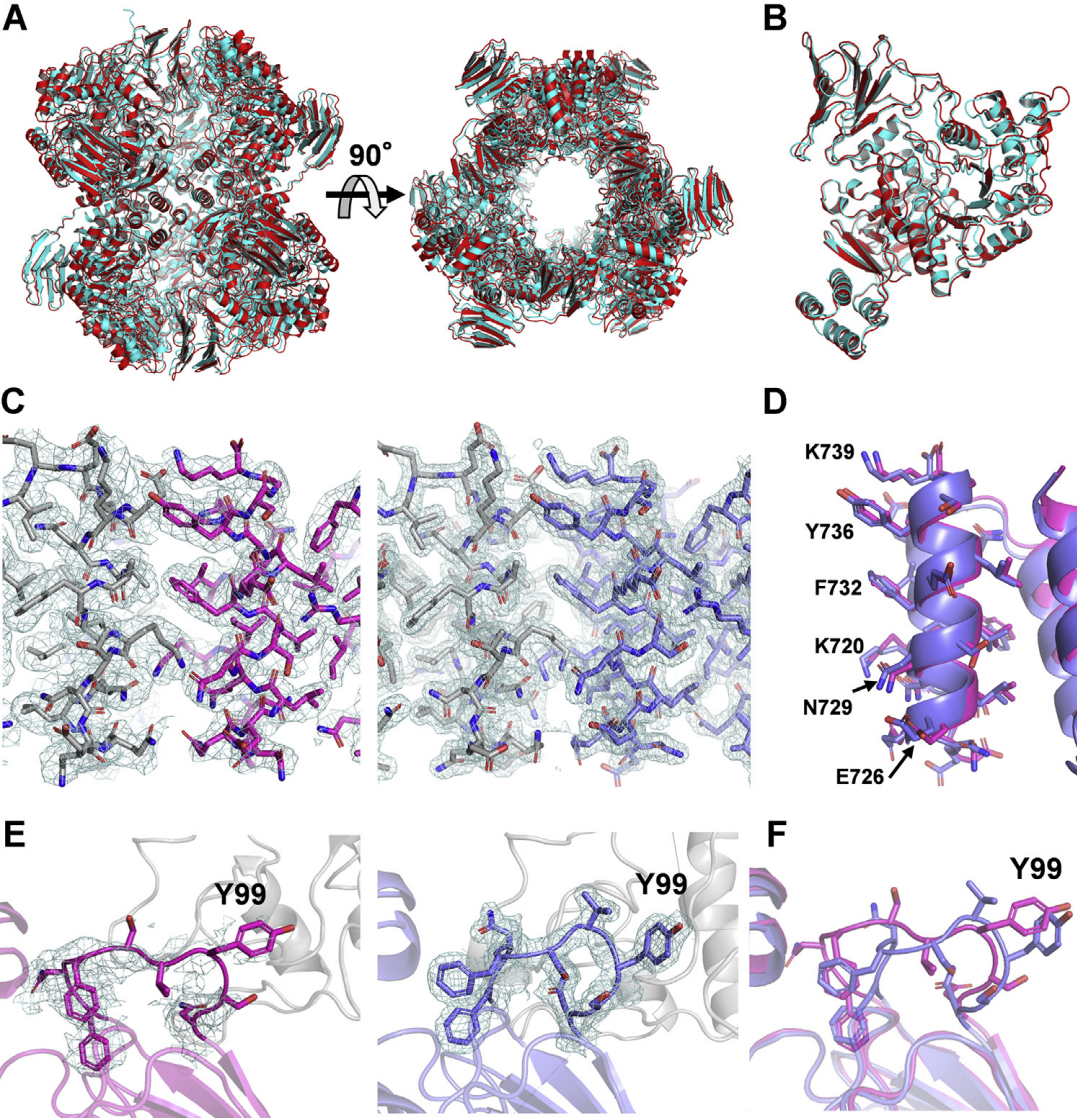
**Comparison with cryo-EM and crystal structures of LlGH31_u1.***A and B*, superimposition of hexamer (*A*) or monomer (*B*) of the cryo-EM structure (*cyan*) and crystal structure (*red*). *C*, cryo-EM map (contoured at 5 *σ*) (*right*) and 2*F*_o_ − *F*_c_ map (contoured at 1 *σ*) (*left*) of the C-terminal α-helix domain. The main and side chains of the domain are shown as *stick* models. *D*, superimposition of the side chains (*stick* model) of the C-terminal α-helix domain in the cryo-EM structure (*magenta*) and crystal structure (*slate blue*). The residues located in the interface are labeled. *E*, cryo-EM map (contoured at 5 *σ*) (*right*) and 2*F*_o_ − *F*_c_ map (contoured at 1 *σ*) (*left*) of the loop in which Y99 is present. *F*, superimposition of the loop where Y99 exists in the cryo-EM structure (*magenta*) and crystal structure (*slate blue*). cryo-EM, cryogenic electron microscopy.

We determined the cryo-EM structure of LlGH31_u1 at 2.73 Å resolution using single-particle analysis (Fig. 4). Table S3 summarizes the statistics of data collection, image processing, and 3D reconstruction steps. The cryo-EM structure revealed the hexameric state of LlGH31_u1, and the overall cryo-EM structure was similar to the crystal structure (Figs. 4, 5 *A* and *B*). The Cα root-mean-square deviation between the cryo-EM and crystal structures is 1.340 Å. In both the cryo-EM and crystal structures, most amino acid residues at the hexamer interface were identical (Figs. 5*C* and S5). The cryo-EM maps for the side chains of the amino acid residues in the C-terminal α-helix domain were well resolved, and most of their conformations were almost identical to those in the crystal structure (Fig. 5, *C* and *D*). At the C-terminal α-helix domain, Val716 and Phe732 hydrophobically interact, and Gln728 forms a hydrogen bond with Lys720 (Fig. S6). By contrast, the local resolutions of insert 1 and insert 2 on A-domain and a loop containing Tyr99 on N-domain (discussed below) are lower than those of the other regions, and the cryo-EM map of these side chains is unclear (Figs. 4*C* and 5*E*). Furthermore, slight structural differences between the crystal and cryo-EM structures were observed (Fig. 5*F*). Although the insertion regions are determined with low *B* factors in the crystal structure (the average *B*-factor values of insert 1 and insert 2 were 30.2 Å^2^ and 33.5 Å^2^, respectively, and those of the other structural components was 33.7 Å^2^), the structural difference may suggest insert 1 and insert 2 flexibility without the crystal packing effect.

### Crystal structures of LlGH31_u1 complexed with ligands

**Figure 6:**
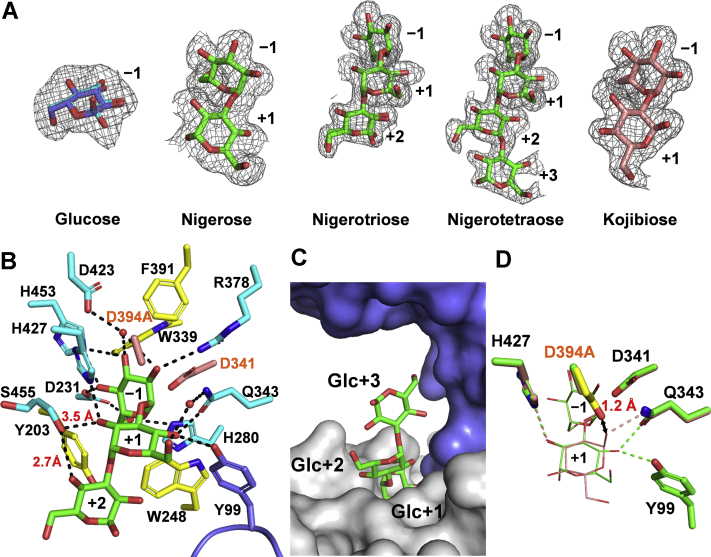
**Ligand–complex structures of LlGH31_u1.***A*, *F*_o_ − *F*_c_ electron density map for ligands (contoured at 2 *σ*). *B*, active sites of D394A-Nig3. Side chains of amino acid residues that interact with nigerotriose are shown as *stick* models. Residues forming hydrogen bonds are colored *cyan*, residues forming a hydrophobic environment are *yellow*, and nigerotriose is *green*. A water molecule is shown as the *red sphere* model and hydrogen bonds are shown as the *dashed line*. *C*, surface model of D394A-Nig4. Nigerotetraose is shown as the *green stick* model, and an adjacent protomer is shown in *slate blue*. *D*, superposition of D394A-Nig2 (*green*) and D394A-Koj2 (*pink*). Side chain of the general acid/base Asp394 (*yellow*) in WT_*P*6_3_22 is also overlaid. Ligands and amino acid residues forming hydrogen bonds with Glc+1 are shown as *thick* and *thin stick* models, respectively. The distance (1.2 Å) between the OD2 of Asp394 and Glc+1 O1 of kojibiose is indicated as the *double arrow*.

First, we determined the crystal structure of WT in complex with glucose (WT-Glc). Asp341 and Asp394, which are located near the C1 atom of the glucose, are assumed to be catalytic nucleophile and acid/base, respectively, similar to other GH31 enzymes (7). In accordance, the structures of inactive mutant D394A, in which the catalytic acid/base D394 was substituted with alanine, and the structure of complexes with nigerose (D394A-Nig2), nigerotriose (D394A-Nig3), nigerotetraose (D394A-Nig4), and kojibiose (D394A-Koj2) were also determined. At the catalytic site of LlGH31_u1, we observed the electron density maps for all ligands. In D394A-Nig3 and WT-Glc, both the α- and β-anomers of the ligand were modeled, while an electron density map for α-nigerose, α-nigerotetraose, and α-kojibiose was observed in D394A-Nig2, D394A-Nig4, and D394A-Koji2, respectively (Fig. 6*A*). Hereafter, the glucose residues bound to subsite −1, subsite +1, subsite +2, and subsite +3 (subsite nomenclature is according to literature (41)) are referred to as Glc−1, Glc+1, Glc+2, and Glc+3, respectively.

In the −1 subsite, the Glc−1 of all ligands (glucose, nigerose, nigerotriose, nigerotetraose, and kojibiose) are accommodated in the active site pocket in almost the same manner (Fig. S7) and recognized by surrounding residues *via* hydrogen bonds and hydrophobic interaction (Fig. 6*B*). In the subsite +1, in addition to three hydrogen bonds with the catalytic site of A-domain, Tyr99 on the N-domain of an adjacent protomer forms a hydrogen bond with the 4-OH group of Glc+1 of nigerooligosaccharides (Fig. 6*B*). The electron densities of two distinct rotamers of Ser455 are observed in some monomers of WT_*P*2_1_ and ligand–complex structures, including D394A-Nig3. One rotamer does not interact with the ligand, but others form hydrogen bonds with the 2-OH of Glc+1 and 4-OH of Glc+2 (Fig. 6*B*). Ser455 may contribute to substrate recognition at subsite +2, and no other amino acid residue forms a hydrogen bond with Glc+2. In D394-Nig4, the Glc+3 of nigerotetraose protrudes from the catalytic pocket (Fig. 6*C*). These findings imply that the substrate recognition of LlGH31_u1 in subsites +2 and +3 is less strict than that in subsites −1 and +1.

In the D394A-Koj2 structure, the Glc+1 adopts the same chair ^4^*C*_1_ conformation as in the D394A-Nig2, but its sugar ring is overturned (Fig. 6*D*). His427 interacts with the 3-OH group of kojibiose Glc+1. Gln343 interacts with the axial 1-OH group of α-kojibiose Glc+1, but Tyr99 on an adjacent subunit does not interact with kojibiose. However, the superimposition of D394A-Koj2 with WT_*P*6_3_22 shows that the estimated distance between the Asp394 and 1-OH of kojibiose was 1.2 Å, indicating a steric hindrance. Although it is unclear how kojibiose binds to the WT catalytic site, β-kojibiose may bind to WT enzyme and/or the glycosidic bond of kojibiose may not be in a suitable position for proton acceptance, resulting in a low turnover.

### Difference in substrate recognition mechanisms between LlGH31_u1 and GH31 α-glucoside hydrolases

**Figure 7:**
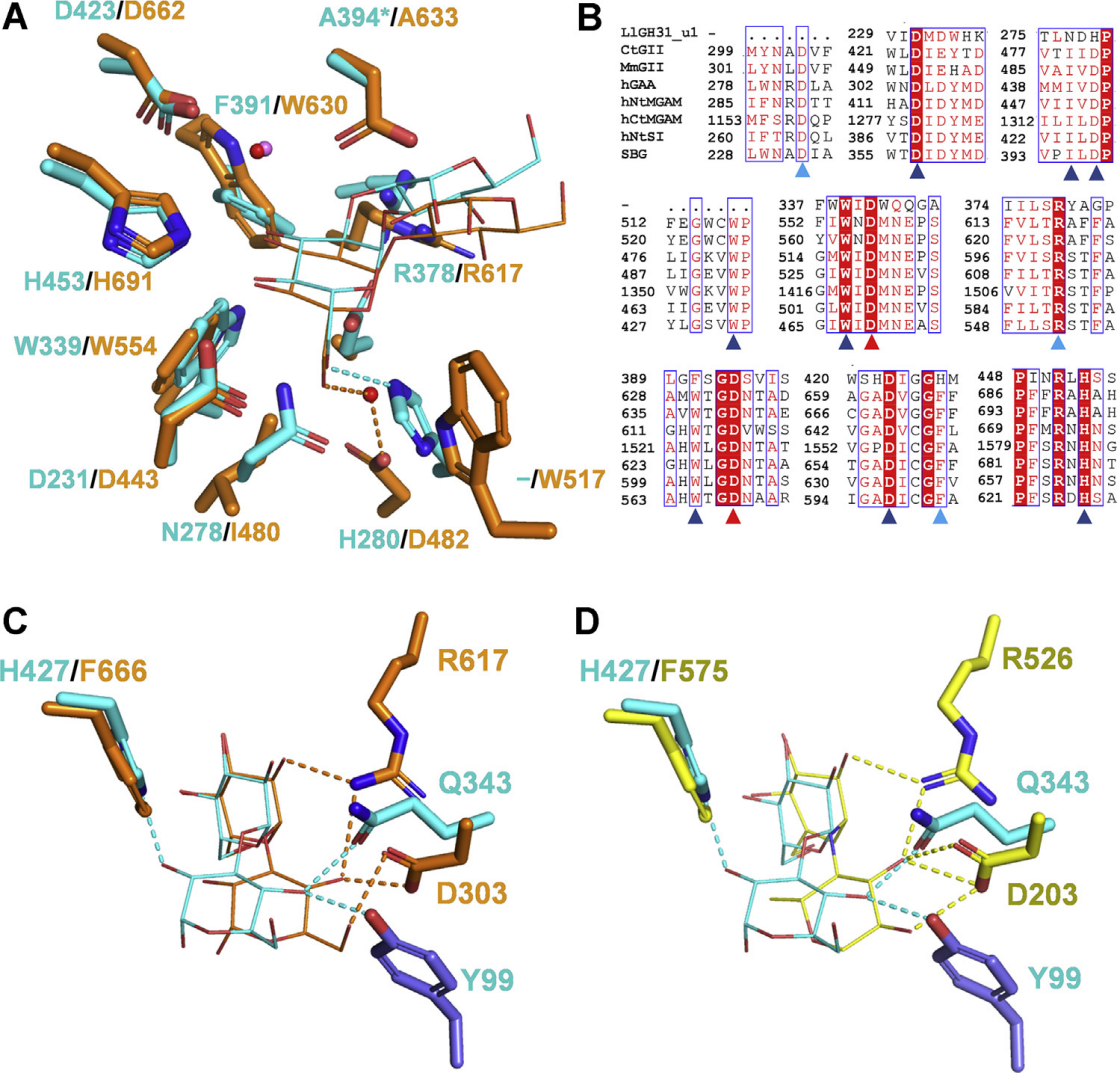
**Comparison of the active sites of LlGH31_u1 with CtGII and NtMGAM.***A*, superimposition of CtGII complexed with nigerose (PDB 5DKZ, *orange*) and D394A-Nig2 (*cyan*). Side chains of amino acid residues at subsite −1 and nigerose are shown as *thick* and *thin stick* models, respectively. Water molecules in CtGII and LlGH31_u1 are shown as *red* and *violet sphere* models, respectively. Hydrogen bonds with the O6 of Glc−1 are shown as *dashed lines*. *Asterisks* indicate an artificially mutated residue (Asp394→Ala). *B*, the amino acid sequence alignment of LlGH31_u1 and eukaryotic α-glucosidases in which structure is determined, *i.e.*, CtGII (GenBank ID, EGS17181.1); MmGII, ER α-glucosidase II from *Mus musculus* (AAC53182.1); hGAA, human lysosomal acid α-glucosidase (CAA68763.1); C-terminal (Ct), and N-terminal (Nt) domains of human MGAM (AAC39568.2); hNtSI, N-terminal sucrase domain of human sucrase-isomaltase (AAT18166.1); SBG, α-glucosidase from sugar beet *Beta vulgaris* (BAM74081.1). *Red* triangles indicate the catalytic residues; *blue* and *light blue* triangles indicate the residues that interact with Glc−1 and Glc+1, respectively. *C*, comparison of subsite +2 in CtGII complexed with nigerose (*orange*) and D394A-Nig2 (*cyan*). Hydrogen bonds between nigerose and the subsite +1 residues are shown as *dashed lines*. Tyr99 side chain of an adjacent protomer in D394A-Nig2 is shown as a *slate blue stick*. *D*, comparison of subsite +1 in NtMGAM complexed with acarbose (*yellow*) and D394A-Nig2 (*cyan*). Acarviosine moiety of acarbose is shown for clarity. Ligands are shown in *thin sticks*. Residues of NtMGAM interacting with α-D-6-deoxy-glucopyranose and those of LlGH31_u1 interacting with nigerose are compared. Hydrogen bonds between substrate and protein are shown as *yellow dashed lines* in NtMGAM and *cyan dashed line* in LlGH31_u1.CtGII, α-glucosidase II from *Chaetomium thermophilum* var. *thermophilum*; NtMGAM, N-terminal maltase domain of human maltase-glucoamylase.

The active site of D394A-Nig2 was compared with CtGII (PDB code 5DKZ). At subsite −1, the glucose recognition mechanisms of LlGH31_u1 and CtGII are similar. At the subsite −1 of CtGII structure, in addition to a pair of catalytic residues, residues that identified Glc−1, Asp443, Trp554, Asp662, and His691 are conserved in LlGH31_u1 (Fig. 7, *A* and *B*). Although Trp630 in CtGII is substituted with Phe391 in LlGH31_u1, the aromatic rings of the residues are located in a similar position and are involved in a hydrophobic environment formation. While the 6-OH group of Glc−1 interacts with Asp482 and Trp517 in CtGII *via* a water molecule, it establishes a direct hydrogen bond with His280 in LlGH31_u1 (Fig. 7*A*). At subsite +1, the amino acid residues involved in glucose recognition differ. Asp303 and Arg617 in CtGII recognize the 4-OH of Glc+1, whereas it is recognized by the Gln343 and Tyr99 of the neighbor protomer in LlGH31_u1. Unlike CtGII, no hydrogen bond with the 2-OH of Glc+1 is observed because the His427 of LlGH31_u1 is substituted with Phe666 in CtGII (Fig. 7*C*).

**Figure 8:**
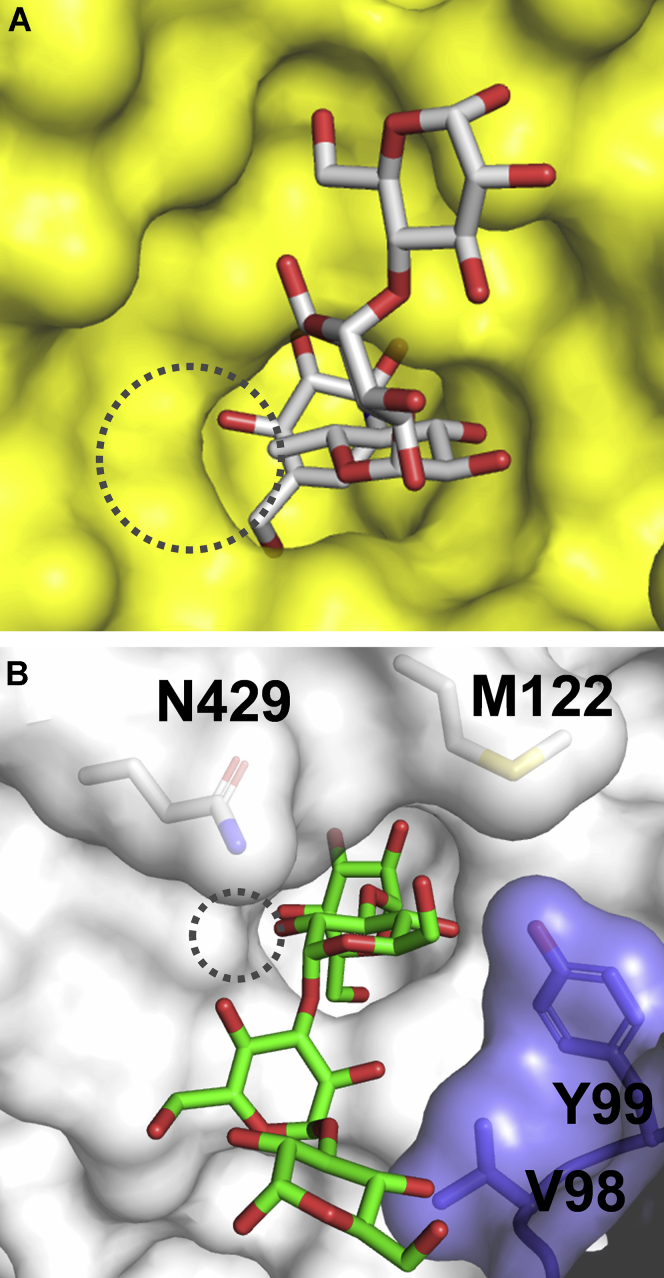
**Surface model of the active sites of NtMGAM and LlGH31_u1.***A*, surface model of NtMGAM in complex with acarbose. Acarbose is shown as a *white stick* model. *B*, surface model of D394A-Nig4. Nigerotetraose is shown as *green stick* models, and adjacent protomer is shown in *slate blue*. *Dotted circles* indicate the space around the methyl group of acarbose and the space around the 2-OH group of Glc+1 of nigerooligosaccharides. NtMGAM, N-terminal maltase domain of human maltase-glucoamylase.

Because no crystal structure of GH31 maltase complexed with maltooligosaccharide substrates is available, the structure of the N-terminal maltase domain of human maltase-glucoamylase (NtMGAM) complexed with inhibitor acarbose (PDB code 2QMJ) (18) was used for comparison to elucidate why LlGH31_u1 has a low activity for maltooligosaccharides. The residues that interact with Glc−1 and Glc+1 of the substrate are conserved in eukaryotic GH31 α-glucosidases, including ER α-glucosidase II and NtMGAM, whose structures have been determined (10, 18) (Fig. 7*D*). In the NtMGAM–acarbose complex, the α-D-6-deoxy-glucopyranose residue adopts the same ^4^*C*_1_ chair conformation as the Glc+1 of nigerose in D394A-Nig2, but the pyranose ring is overturned. If maltose interacts with acarbose, Gln343, and Tyr99 in a similar orientation, residues that form hydrogen bonds with a 4-OH group of Glc+1 of nigerose may not prevent maltose from binding. A space exists surrounding the methyl group of acarbose in the NtMGAM–acarbose complex structure, which is probably capable of accepting the 6-OH group of Glc+1 of maltooligosaccharides (Fig. 8*A*). However, because LlGH31_u1 lacks additional space surrounding the 2-OH group of Glc+1 of nigerooligosaccharides (Fig. 8*B*), the 6-OH group of maltose may have difficulty fitting into the catalytic pocket. Moreover, compared with the NtMGAM–acarbose complex, Met122 and Asn429 in LlGH31_u1 may inhibit acarbose binding (Fig. 8, *A* and *B*). A similar steric hindrance for acarbose is also observed in the α-subunit of ER α-glucosidase II (10). These findings support the result that LlGH31_u1 is substantially less active for maltooligosaccharides.

### Function of LlGH31_u1 Tyr99

Structural analysis suggested that the Tyr99 on N-domain is involved in the formation of subsite +1 of an adjacent protomer. However, the cryo-EM structure suggested the flexibility of this residue (Fig. 5*E*), and this tyrosine residue is not strictly conserved among LlGH31_u1 homologs, including CmGH31_u1 (Fig. S8). Therefore, we constructed a valiant Y99F in which Tyr99 is substituted with phenylalanine to determine whether Tyr99 affects LlGH31_u1 activity. Y99F was expressed in *E. coli* and purified in the same manner as the WT enzyme, and its activities for various substrates were determined. Contrary to our expectations, Y99F exhibits the same substrate preference as the WT enzyme (nigerose >> kojibiose > maltose > isomaltose) but is inactive against trehalose and sucrose (Table 1). The *K*_m_ values of the LlGH31_u1_Y99F mutant for nigerose and nigerotriose were 12 ± 2 mM and 2.0 ± 0.2 mM, respectively, which were nearly identical to those of WT. The *k*_cat_ values for nigerose and nigerotriose were 5.2 ± 0.4 s^−1^ and 4.9 ± 0.4 s^−1^, respectively, and are approximately two times lower than that of WT. By contrast, the *k*_cat_ value for pNP-Glc, 59 ± 0.8 s^−1^, was two times higher than that of WT (Table 2). Thus, it was proposed that Tyr99 affects the turnover of the hydrolytic reaction rather than the substrate specificity. However, further analysis is required to elucidate the role of Tyr99.

### Estimation of the physiological role of LlGH31_u1 and its homologs

Although some bacterial species degrade eukaryotic *N*-glycans, mature proteins lack nigerose units on the nonreducing ends of *N*-glycans because the α-1,3-glucosidic moiety is cleaved in ER (37, 42). Therefore, eukaryotic *N*-glycan may not be a natural substrate of LlGH31_u1, unlike ER α-glucosidase II. One possible source of the substrate of LlGH31_u1 is mutan, which is an exopolysaccharide that contains α-1,6 and α-1,3 bonds and is synthesized by three different glucansucrases belonging to the GH70 family in *Streptococcus mutans* (43). The genome of *S. mutans* encodes *GH31_u1* and may be involved in mutan metabolism. Some lactic acid bacteria also synthesize exopolysaccharides, which contain a major α-1,6 linkage and a minor α-1,3 linkage (44, 45, 46). However, there has been no report on the synthesis of glucans with α-1,3 linkage from *Lactococcus* species, and there is no gene estimated as GH70 glucansucrase in *L. lactis* genome.

α-1,3-Glucan produced by fungi is another potential natural source of GH31_u1 substrate. The cell wall of filamentous fungi contains α-1,3-glucan, which has an α-1,3-glucosidic linkage and partial α-1,4 linkage as one of the principal components (47). Based on the result that CmGH31_u1 exhibited high substrate specificity to nigerose, fungal GH31_u1 homologs may be involved in the metabolism of α-1,3-glucans. Because the hydrolytic activity of LlGH31_u1 for nigeran was not detected, GH31_u1 may require cooperation with an endo-type enzyme to degrade polysaccharides. GH71 proteins are known as fungal endo-α-1,3-glucanases, and *C. millitalis* also has a gene encoding a GH71 protein. In addition, *Talaromyces verruculosus* has a gene for GH31_u1 fused with the GH71 domain (GenBank, KUL90319.1). Therefore, fungal GH31_u1 enzymes may be involved in the degradation of cell wall α-1,3-glucan in cooperation with GH71 enzymes.

**Figure 9:**
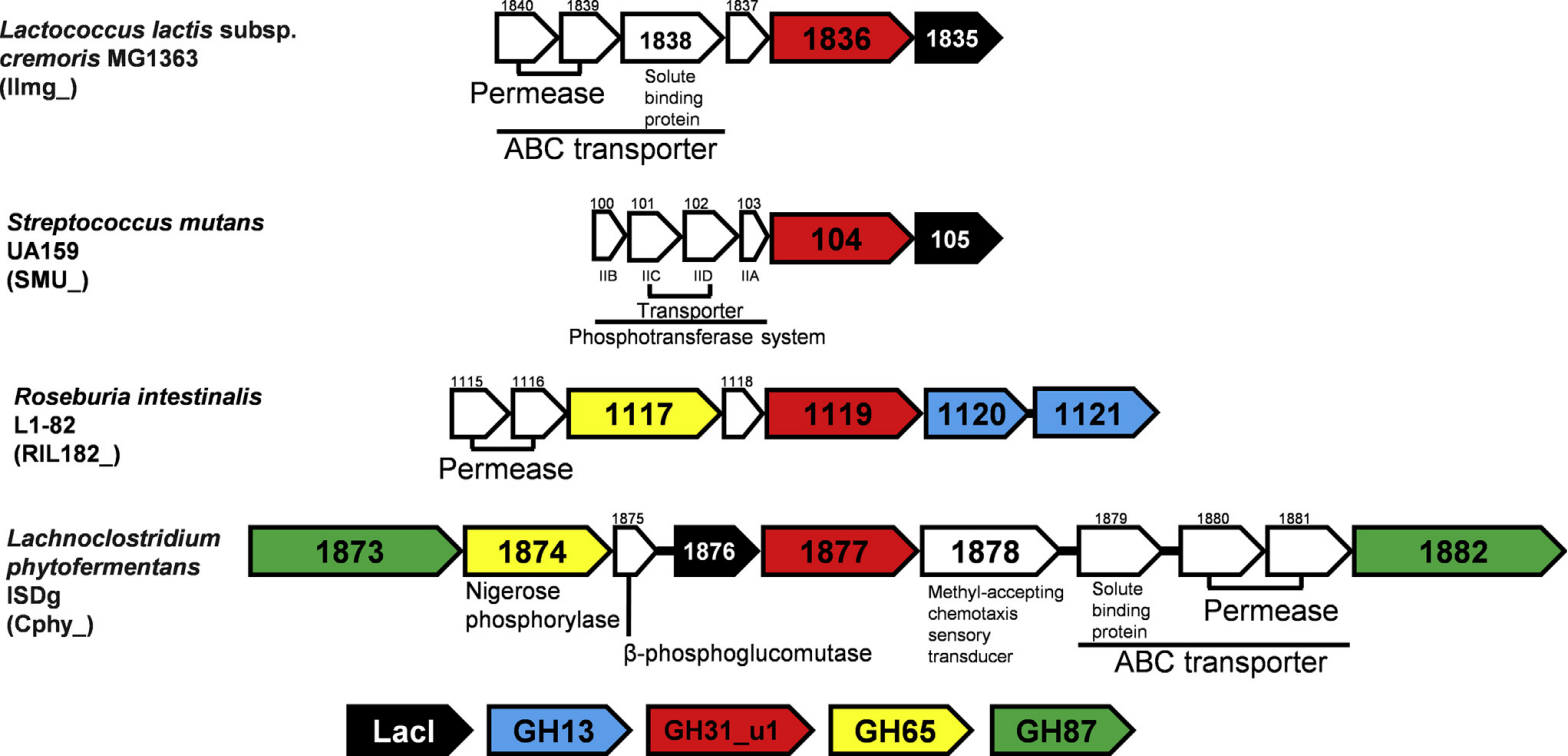
**Gene clusters, including *GH31_u1*, in bacteria.** Gene clusters containing genes for GH31_u1 proteins from *Lactococcus lactis* subsp. *cremoris* MG1363 (GenBank ID, CAL98407.1), *Streptococcus mutans* UA159 (AAN57886.1), *Roseburia intestinalis* L1-82 (VCV21248.1), *Lachnoclostridium phytofermentans* ISDg (ABX42246.1), and *Bacteroides cellulosilyticus* WH2 (CCP32040.1) are shown. Open reading frames are shown as *arrows*, and colors are listed below the gene clusters. LacI indicates the LacI-like transcription regulator.

Nigerose phosphorylase from *L. phytofermentans* (Cphy1874), belonging to GH65, has high substrate specificity for nigerose. In the genome of *L. phytofermentans*, a putative GH87 endo-α-1,3-glucanase is located near the GH65 nigerose phosphorylase, and its participation in nigerose degradation is proposed (36). In addition, *GH31_u1* (locus tag Cphy_1877) is located in the same gene cluster as nigerose phosphorylase, along with genes for ABC transporter proteins and LacI family transcription regulators (Fig. 9). In the genome of *L. lactis*, LlGH31_u1 is also surrounded by the genes for ABC transporter proteins and LacI family transcription regulator. *S. mutans* has a gene cluster similar to that of *L. lactis*; however, it has the genes for phosphotransferase system instead of the ABC transporter system (48, 49). Other bacterial species harboring *GH31_u1* possess various GHs and sugar utilization proteins genes in the vicinity of *GH31_u1*; therefore, GH31_u1 may be involved in various degradation systems for saccharides in widespread species. *L. lactis* is isolated from various sources, including dairy products, fermented foods, plants, and soil (50). *L. lactis* may absorb and use oligosaccharides, which might be derived from polysaccharides produced by other organisms, using LlGH31_u1. However, LlGH31_u1 may require cooperation with other enzymes, at least with an endo-type glycoside degradation enzyme, to degrade polysaccharides with α-1,3-glucosidic linkage; therefore, further study is required to clarify the entire pathway in which LlGH31_u1 is involved.

## Conclusion

We found that microbial GH31 enzymes displayed more strict specificity to α-1,3-glucosides than other characterized GH31 enzymes. X-ray crystallographic and cryo-EM structural analyses revealed that LlGH31_u1 forms a hexamer, and the C-terminal α-helix domain, which has not been observed in other GH31 enzymes, is involved in hexamer formation. Moreover, the residues forming subsite +1, including the Tyr99 residue on an adjacent protomer, of LlGH31_u1 are different from those of the other GH31 α-glucoside hydrolases and are important for the mechanism of the strict recognition of α-1,3-glucosidic linkages. This study suggests that LlGH31_u1 and its homologs are involved in the degradation of nigerooligosaccharides in various microbial species, and further studies are needed to clarify the entire pathway of polysaccharide degradation in which GH31_u1 enzymes are involved.

## Experimental procedures

### Chemicals and strains

*p*-Nitrophenyl α-D-galactopyranoside and GlcNAc-β-1,3-GalNAcα-pNP were purchased from Tokyo Chemical Industry Co, Ltd *p*-Nitrophenyl α-L-fucopyranoside, pNP-α-Glc, and *p*-nitrophenyl α-D-mannopyranoside were obtained from Merck. *p*-Nitrophenyl *N*-acetyl-α-D-glucopyranoside, *p*-nitrophenyl α-L-rhamnopyranoside, *p*-nitrophenyl α-D-xylopyranoside, nigerose, and nigeran were purchased from Carbosynth. GalNAcα-pNP was obtained from Cayman Chemical, and nigerotriose and nigerotetraose were purchased from Megazyme. Maltose, maltotriose, and maltotetraose were obtained from Hayashibara Co *E. coli* DH5α and BL21 (DE3) were used for DNA manipulation and protein expression, respectively.

### Phylogenetic analysis

The extraction of accession numbers registered on the CAZy database, download of the corresponding amino acid sequences from National Center for Biotechnology Information, and pruning of extra domains was performed using the SACCHARIS program (28). These sequences were then clustered at 70% sequence similarity using CD-HIT to remove redundancy (51, 52). The sequence alignment of GH31 catalytic domains was performed using the MUSCLE algorithm, and the phylogenetic tree was built *via* the maximum likelihood method using MEGA (53). The phylogenetic tree was visualized using iTOL (54).

### Plasmid construction and site-directed mutagenesis

The absence of a signal peptide of LlGH31_u1 and CmGH31_u1 was evaluated using SignalP 5.0 (http://www.cbs.dtu.dk/services/SignalP/). The DNA fragment encoding LlGH31_u1 (llmg_1836, GenBank CAL98407.1) was amplified from *L. lactis* subsp. *cremoris* MG1363 using colony-directed polymerase chain reaction (PCR) with Ex Taq DNA polymerase (TaKaRa). The DNA fragment encoding CmGH31_u1 was amplified from the cDNA of *C. militaris* NBRC 103752 (NITE BioResource Center). The resultant DNA fragments were digested with NdeI and NotI (for LlGH31_u1) or NdeI and HindIII (for CmGH31_u1) and then ligated into a pET28a vector (Merck) digested using the same restriction enzymes. Site-directed mutagenesis was performed *via* inverse PCR with the desired primers using pET28a expression plasmid harboring DNA encoding LlGH31_u1 as a template. All single amino acid substitution mutants were generated according to literature (55). All primers used are listed in Table S4. All the constructed plasmids were confirmed *via* DNA sequencing.

### Recombinant expression and purification

*E. coli* BL21 (DE3) harboring the desired plasmid was cultured at 37 °C to an absorbance (600 nm) of 0.8 in 1 L Luria–Bertani medium containing 50 μg/ml kanamycin. After cooling the medium to 20 °C on ice, protein expression was induced by adding 0.05 and 0.5 mM IPTG at 20 °C for 20 h for WT LlGH31_u1 and the other expression constructs, respectively. The cells were harvested *via* centrifugation for 10 min (4 °C 5000*g*) and stored at −20 °C. The cell pellet was resuspended in 50 mM sodium phosphate buffer (pH 8.0) containing 300 mM NaCl and 20 mM imidazole. After resuspension, the cells were disrupted *via* ultrasonication and then centrifuged to remove insoluble materials. Cell lysate supernatant was loaded onto a Ni-NTA Agarose (Qiagen) column, and the unbound proteins were washed with the same buffer. Proteins were eluted with the same buffer containing 250 mM imidazole and then concentrated *via* ultrafiltration using 30K Amicon Ultracentrifugal units. Gel filtration chromatography was performed using the ÄKTA explorer 10S system (GE Healthcare). LlGH31_u1 was applied onto Superdex 200 Increase 10/300 column for protein purification or HiLoad 16/60 Superdex 200 prep grade column for molecular weight determination and eluted by 1.5 column volumes of 20 mM sodium phosphate buffer (pH 7.0) containing 300 mM NaCl. Protein purity was confirmed using sodium dodecyl sulfate-polyacrylamide gel electrophoresis.

### Enzyme assays

The hydrolytic activity toward various *p*-nitrophenyl (pNP) glycosides was measured in 50 μl reaction mixtures containing 40 μg/ml of LlGH31_u1 or CmGH31_u1, 0.5 mM of a substrate, and 20 mM sodium phosphate buffer (pH 7.0) at 30 °C. To examine the effect of pH on hydrolytic activity, reaction mixtures containing LlGH31_u1 (30 μg/ml) or CmGH31_u1 (34 μg/ml) and 0.5 mM pNP-α-Glc were prepared with McIlvaine (sodium citrate–phosphate) buffer at pH 3.5 to 8.0 or in glycine–HCl buffer at pH 9.0 to 10. The mixtures were incubated for 5 min at 30 °C. The effect of temperature on the hydrolytic activity was examined using 50 mM sodium phosphate buffer (pH 7.0) containing 0.5 mM pNP-α-Glc. The mixtures were incubated at temperatures ranging from 25 °C to 55 °C. The pH stability was tested by incubating 500 μg/ml of LlGH31_u1 for 24 h in McIlvaine buffer at pH 3.5 to 8.0 or in glycine–HCl buffer at pH 9.0 to 10. The thermostability was tested by incubating 300 μg/ml of LlGH31_u1 at 4 °C to 45 °C for 30 min. The residual activities for pH stability and thermostability were measured using 30 μg/ml of LlGH31_u1 and 50 mM phosphate buffer (pH 7.0) containing 0.5 mM pNP-α-Glc at 30 °C. All reactions above were terminated by adding 100 μl of 1 M Na_2_CO_3,_ and the amount of released pNP was quantified by measuring absorption at 405 nm.

To examine the activity toward disaccharides, the reaction mixture containing 20 mM sodium phosphate buffer, 10 mM disaccharides (trehalose, kojibiose, nigerose, maltose, isomaltose, or sucrose), and 0.1 mg/ml purified LlGH31_u1 was incubated at 30 °C for 1 h and analyzed by TLC using Silica Gel 60 F_254_ TLC plates (Merck). TLC plates were developed using a solvent with 1-butanol/ethanol/water (10:5:2, vol/vol) and sprayed with 10% sulfuric acid in methanol and then baked. For the quantitative analysis of GH31_u1 enzyme hydrolytic activity against α-glucosides, the reaction was terminated by adding the same amount of 0.5 M sodium carbonate (pH 10.0), and the liberated glucose was quantified by the glucose oxidase–peroxidase method using Glucose CII-test Wako (Wako Pure Chemicals). To determine kinetic parameters, initial velocities at five or six concentrations for each substrate were measured and fitted to the Michaelis−Menten equation using Kaleida Graph software (Synergy Software). The reaction was performed using various concentrations of LlGH31_u1 (62.0 nM for nigerose and nigerotetraose, 32.0 nM for nigerotriose, 611 nM for maltose, 1.55 × 10^3^ nM for maltotriose, 3.10 × 10^3^ nM for maltotetraose, 310 nM for kojibiose, and 49.0 nM for pNP-Glc; all concentrations were calculated as a monomer) or CmGH31_u1 (620 nM for nigerose and nigerotriose and 124 nM for pNP-α-Glc) in 50 mM sodium phosphate buffer (pH 7.0 and pH 6.0 for LlGH31_u1 and CmGH31_u1, respectively) containing 50 mM sodium chloride. Substrate concentrations were as follows: 1 to 16 mM for pNP-α-Glc, 1 to 20 mM for nigerose, 0.5 to 10 mM for nigerose and nigerotetraose, 1 to 30 mM for kojibiose, 1 to 50 mM for maltose, and 5 to 100 mM for maltotriose and maltotetraose.

### X-ray crystallography

The purified protein was concentrated to 15 mg/ml in 10 mM Hepes (pH 7.0) *via* ultrafiltration and crystallized at 20 °C using the hanging-drop vapor diffusion method, in which 1.0 μl protein solution was mixed with an equal volume of a crystallization reservoir solution. Initial crystallization screening was performed using Crystal Screen, Crystal Screen 2, PEG/Ion Screen, and PEG/Ion 2 Screen kits (Hampton Research). Crystallization trials using PEG/Ion Screen and PEG/Ion 2 Screen yielded snowflake-shaped crystals in several conditions. Additive screening was performed using an Additive Screen kit (Hampton Research), and crystal form 1 was obtained using a crystallization solution containing 16% (w/v) polyethylene glycol (PEG) 3350, 200 mM sodium citrate buffer (pH 7.0), and 3% (w/v) xylitol. The crystal form 2 of native LlGH31_u1, D394A, and selenomethionine (SeMet)-substituted LlGH31_u1 were obtained using a crystallization solution containing 13% to 7% (w/v) PEG 3350 and 400 mM sodium citrate buffer (pH 7.0). The crystal was soaked for 45 min in the reservoir solution containing 100 mM glucose to determine LlGH31_u1 in complex with glucose. To determine the structures of LlGH31_u1 in complex with the other ligands, enzymes were cocrystallized using a reservoir solution containing 10 mM ligands. Crystals were cryoprotected with the reservoir solution supplemented with ethylene glycol at a final concentration of 22% (v/v) and then flash-frozen in liquid nitrogen or flash cooled to 100 K in a nitrogen gas stream. Diffraction data were collected at the BL5A and AR-NW12A beamlines (Photon Factory).

Data were processed using XDS (56). The initial phase was calculated using the single-wavelength anomalous dispersion dataset of SeMet-substituted LlGH31_u1 *via* the Crank2 program in the CCP4 suite (57). The structures of WT_*P*2_1_ and WT_*P*6_3_22 were solved by the molecular replacement method using MOLREP (58) with the coordinates of the refined SeMet-LlGH31_u1 as the search models. Refinement and manual model building were performed using REFMAC5 (59) and COOT (60), respectively, and the models were validated using MolProbity (61).

### Cryo-EM sample preparation and data acquisition

A 3 μl sample of LlGH31_u1 (1.13 μM, calculated as a hexamer) in 20 mM sodium phosphate buffer (pH 7.0) containing 300 mM NaCl was applied to a holey carbon grid (Quantifoil, Cu, R1.2/1.3, 300 mesh) rendered hydrophilic by a 30-s glow-discharge in air at 11 mA current with PIB-10. The grid was blotted for 5 s (blot force 15) at 18 °C and 100% humidity and flash-frozen in liquid ethane using Vitrobot Mark IV (FEI).

A total of 995 micrographs were acquired by two cryo-EM sessions of Talos Arctica (FEI) microscope operating at 200 kV in the nanoprobe mode using EPU software for automated data collection. For both sessions, the movie frames were collected by 4 k × 4 k Falcon 3 direct electron detector in electron counting mode at a nominal magnification of 1,200,00 × , which yielded a pixel size of 0.88 Å/pixel. Fifty movie frames were recorded at an exposure of 1.00 electrons per Å^2^ per frame, corresponding to a total exposure of 50 e^−^/Å^2^. The defocus steps used were –0.8, –1.2, –1.6, and –2.0 μm.

### Cryo-EM data processing

Movie frames were aligned, dose-weighted, and averaged using MotionCor2 (62) (the version implemented in RELION3.1 (63)) on 5 × 5 tiled frames with a B-factor of 200 applied to correct for beam-induced specimen motion and account for radiation damage using an exposure-dependent filter. The micrographs whose total accumulated motions were >60 Å were discarded. The nonweighted movie sums were used for contrast transfer function (CTF) estimation with the program Gctf (64) (512-pixel box size, 30 Å minimum and 4 Å maximum resolution, and 0.10 amplitude contrast), whereas the dose-weighted sums were used for all subsequent steps of image processing. First, the images whose CTF max resolutions were better than 5.5 Å were selected. The particles were collected using SPHIRE crYOLO with a generalized model (65, 66) using a selection threshold of 0.1. The subsequent processes of 2D classification, *ab initio* reconstruction, 3D classification, 3D refinement, CTF refinement, Bayesian polishing, and local resolution estimation were conducted using RELION3.1. The details of the processes are described in the Supporting information (Supplementary Methods, Figs. S9, and S10).

For calculating the global resolution estimation after each 3D refinement, the gold standard FSC resolution with 0.143 criterion (67) was used, including the phase randomization to account for the possible artifactual resolution enhancement caused by solvent mask (68). The model-to-map FSC resolution with 0.5 criterion was calculated using phenix.mtriage (69). To visualize the output 2D/3D images, UCSF Chimera and e2display.py of EMAN2 (70) were used. The ctflimit function (71) implemented in SPARX/SPHIRE (66, 72) was used to calculate the smallest box size that ensures no CTF aliasing in the reciprocal space up to an expected resolution for the maximum defocus value of the dataset.

## Data availability

The nucleotide sequence of *CmGH31_u1* was submitted to the DDBJ/EMBL/GenBank databases under the accession number LC660181. The atomic coordinates and structure factors of WT_*P*2_1_, WT_*P*6_3_22, WT-Glc, D394A-Nig2, D394A-Nig3, D394A-Nig4, and D394A-Koj2 have been deposited in the Worldwide Protein Data Bank (wwPDB, http://wwpdb.org/) under the accession codes 7WJ9, 7WJA, 7WJB, 7WJC, 7WJD, 7WJE, and 7WJF, respectively. The cryo-EM map and the atomic coordinate of LlGH31_u1 determined using cryo-EM have been deposited in the Electron Microscopy Data Bank (https://www.ebi.ac.uk/pdbe/emdb/) and wwPDB, respectively, with the accession codes EMD-32571 and 7WLG.

## Supporting information

This article contains supporting information (8, 30, 36, 37, 53, 54, 73, 74, 75, 76, 77, 78, 79, 80, 81).

## Conflict of interest

The authors have no conflicts of interest to declare.
