# Supplementary material for: Structural basis of the strict specificity of a bacterial GH31 α-1,3-glucosidase for nigerooligosaccharides
Source: J Biol Chem. 2022 Mar 12;298(5):101827. doi: 10.1016/j.jbc.2022.101827 (PMC9061262; doi:10.1016/j.jbc.2022.101827)
Supplement: Supporting information [file mmc1.pdf]

Supporting information

Structural basis of the strict specificity of a bacterial GH31 α-1,3-glucosidase for nigerooligosaccharides

Marina Ikegaya (池谷 真里奈)[1], Toshio Moriya (守屋 俊夫)[2], Naruhiko Adachi (安達 成彦)[2], Masato Kawasaki (川崎 政人)[2,3], Enoch Y. Park (朴 龍洙)[1,4], and Takatsugu Miyazaki (宮崎 剛亜)[1,4*]

[1] Department of Bioscience, Graduate School of Science and Technology, Shizuoka University, 836 Ohya, Suruga-ku, Shizuoka, 422-8529, Japan.
[2] Structural Biology Research Center, Institute of Materials Structure Science, High Energy Accelerator Research Organization (KEK), 1-1 Oho, Tsukuba, Ibaraki 305-0801, Japan.
[3] Department of Materials Structure Science, School of High Energy Accelerator Science, The Graduate University of Advanced Studies (Soken-dai), 1-1 Oho, Tsukuba, Ibaraki 305-0801, Japan.
[4] Research Institute of Green Science and Technology, Shizuoka University, 836 Ohya, Suruga-ku, Shizuoka, 422-8529, Japan.

*Correspondence: Takatsugu Miyazaki, Research Institute of Green Science and Technology, Shizuoka University, 836 Ohya, Suruga-ku, Shizuoka, 422-8529, Japan; E-mail: miyazaki.takatsugu@shizuoka.ac.jp

List of materials included

Supplementary Methods
Table S1. Kinetic parameters of GH31 and GH65 enzymes for nigerose and maltose.
Table S2. Data collection and refinement statistics.
Table S3. Data collection, processing, and reconstruction statistics for cryo-EM.
Table S4. Oligonucleotide primers used in this study.
Figure S1. Phylogenetic tree of LlGH31_u1 proteins.
Figure S2. Effects of pH and temperature on the hydrolytic activity of LlGH31_u1.
Figure S3. Effects of pH and temperature on the hydrolytic activity of CmGH31_u1.
Figure S4. Comparison of the hexameric structures of GH31 enzymes.
Figure S5. Amino acid residues on the interface of LlGH31_u1 hexamer.
Figure S6. Interface residues of C-terminal α-helix domain.
Figure S7. Superimposition of ligand-free and ligand-complex structures.
Figure S8. Residues conserved among GH31_u1 homologs.
Figure S9. Cryo-EM data processing workflow.
Figure S10. Validation of cryo-EM single-particle analysis.
References

Supplementary Methods

The dataset of the 1st cryo-EM session (Subset A) was processed as follow (Supplementary Fig. S9). A stack of 139,450 particle images was extracted from 470 dose-weighted sum micrographs while rescaling to 3.52 Å/pixel with 64-pixel box size, and subjected to 3 successive runs of reference-free 2D classification (200 expected classes, 214, 187, and then 159 Å mask diameter) while discarding 2D classes whose average images showing obvious non-protein features after each 2D classification. From the 3rd run, the 68,096 particles corresponding to the best 25 classes that displayed secondary-structural elements and multiple views of LlGH31_u1 were selected for *ab initio* reconstruction (asymmetry, single expected class, 187 Å mask diameter), and also the 127,990 particles (41 classes) were selected for the following 3D classification with a more relaxed selection criterion. D3 symmetry was imposed on the generated *ab initio* map, and then the volume was low-pass filtered to 30 Å and used as an initial model for 3D classification (4 expected classes, 214 Å mask diameter). The map of the 3D class with the highest resolution was low-pass filtered to 15 Å, and used as an initial model for the subsequent 3D refinement (D3 symmetry, 214 Å mask diameter, with padding) with the 36,014 selected particles.

The refined map was rescaled to 1.10 Å/pixel with 320-pixel box size, low-pass filtered to 15 Å, and used as an initial model for the subsequent 3D refinement. Accordingly, the selected particle images were also re-centered and re-extracted using the same rescale settings. The particle images which became duplicated as the result of alignments and not fully inside of the micrograph boundary anymore due to changing the box size were excluded. 33,940 selected particles were 3D auto-refined (D3 symmetry, 334 Å mask diameter, no padding) twice, the 1st run without 3D mask, the 2nd with a soft-edged 3D mask created from the output of 1st run (5-pixel extension, 10-pixel soft cosine edge). Then, local 3D refinement was executed with same settings including 3D mask. To refine the per-particle defocus, beam tilt, and beam-induced motion corrections, the cycle of CTF refinement (1) and Bayesian polishing (2) was repeated 3 times. The number of repeats ensured that there were no further improvements. To measure the degree of improvement, local 3D refinement (D3 symmetry, 334 Å mask diameter, no padding, solvent-flattened FSCs enabled) with the same 3D mask was used after each CTF refinement and Bayesian polishing step. The 3D refinement after the 3rd polish step was repeated starting from global search and obtained 3.03 Å resolution map.

The dataset of the 2nd cryo-EM session (Subset B) was processed likewise. The details are given in Supplementary Fig. S9. The differences from the processing of subset A were (i) the *ab initio* reconstruction was skipped, and the D3-imposed *ab initio* map generated with subset A was used as an initial model for the first 3D classification of subset B, (ii) the cycle of CTF refinement and Bayesian polishing was repeated 4 times and (iii) the global 3D refinement after the 4th polish step was not repeated. The 3D refinement after the 4th polish step generated 3.14 Å resolution map.

The particles from the both subsets were again re-centered and re-extracted with 512-pixel box size, followed by removal of duplicated and out-of-bounds particles, and were merged. Accordingly, the density map with the highest resolution so far was also rescaled with the same settings, low-pass filtered to 15 Å, and used as an initial model for the subsequent 3D refinement. 63,338 selected particles were 3D auto-refined (D3 symmetry, no padding) twice, the 1st run using 224 Å mask diameter without 3D mask, the 2nd using 336 Å mask diameter with a soft-edged 3D mask created from the output of 1st run (no pixel extension, 10-pixel soft cosine edge). Then, local 3D refinement was executed (without low-pass filter, D3 symmetry, 428 Å mask diameter, no padding, solvent-flattened FSCs enabled) with the same 3D mask, and obtained 3.22 Å structure. The 3 cycles of CTF refinement and Bayesian polishing steps were executed and generated the 2.78 Å structure. As before, local 3D refinement (without low-pass filter, D3 symmetry, 428 Å mask diameter, no padding, solvent-flattened FSCs enabled) with the same 3D mask was used after each CTF refinement and Bayesian polishing step.

To improve homogeneity of the particle stack, no-alignment 3D classification was conducted by setting expected classes to 2 and regularization parameter T to 128 (without low-pass filter, D3 symmetry, 428 Å mask diameter, no padding) with the same 3D mask, and selected 44,606 particles by choosing the 3D class with higher resolution. Subsequently, 3D refinement (low-pass filter cutoff 15 Å, D3 symmetry, 336 Å mask diameter, no padding) and local 3D refinement (without low-pass filter, D3 symmetry, 428Å mask diameter, no padding, solvent-flattened FSCs enabled) with the same 3D mask were conducted. The 2 cycles of CTF refinement and Bayesian polishing steps were executed as before. Using the outputs of 2nd Bayesian polishing step, 3D refinement (low-pass filter cutoff 15 Å, D3 symmetry, 336 Å mask diameter, no padding), and then, local 3D refinement (without low-pass filter, D3 symmetry, 428Å mask diameter, no padding, solvent-flattened FSCs enabled) with the same 3D mask generated the final result of 2.73 Å resolution, while the model-to-map FSC resolution was 2.83 Å (Supplementary Fig. S10B) . The local resolution of the final map was estimated using the algorithm implemented in the RELION-3.1.

Table S1. Kinetic parameters of GH31 and GH65 enzymes for nigerose and maltose.

Enzyme	Nigerose			Maltose			Reference
	K_m (mM)	k_{cat} (s^{-1})	k_{cat}/K_m (s^{-1} mM^{-1})	K_m (mM)	k_{cat} (s^{-1})	k_{cat}/K_m (s^{-1} mM^{-1})	
LlGH31_u1	9.2 ± 0.6	9.7 ± 0.3	1.1	36 ± 1.9	0.79 ± 0.02	0.022	This study
LJAG31[a]	1.79 ± 0.07	78.6 ± 0.7	44	30.8 ± 1.6	123 ± 3	4.0	(3)
SpGII[b]	2.13 ± 0.06	0.102 ± 0.001	0.048	18.0 ± 0.1	0.241 ± 0.007	0.013	(4)
α-Glucosidase from *Aspergillus niger*	12	7.64	0.64	0.75	144	192	(5)
Rabbit GAA[c]	16.0	179.5	11	6.3	179.5	28	(6)
human NtMGAM[d]	27.1 ± 2.6	120.9 ± 3.7	4.5	8.7 ± 1.3	110.2 ± 3.8	13	(7)
human CtMGAM[e]	35.2 ± 3.6	96.0 ± 3.3	2.7	2.6 ± 0.6	133.9 ± 4.3	52	(7)
human ntSI[f]	44.4 ± 6.4	24.8 ± 1.3	0.56	11.1 ± 1.5	18.3 ± 0.6	1.6	(7)
human ctSI[g]	63.6 ± 13.0	11.0 ± 1.0	0.17	4.2 ± 1.4	11.4 ± 0.8	2.7	(7)
Cphy1874[h]	1.7 ± 0.2	67 ± 2.4	39	Not active	Not active	-	(8)

[a]GH31 α-(1→3)-glucosidase form *Lactobacillus johnsonii*; [b] α-Glucosidase II from *Schizosaccharomyces pombe*; [c]Acid α-glucosidase; [d]N-terminal domain of maltase-glucoamylase; [e]C-terminal domain of maltase-glucoamylase; [f]N-terminal domain of sucrase-isomaltase; [g]C-terminal domain of sucrase-isomaltase; [h]GH65 nigerose phosphorylase form *Lachnoclostridium phytofermentans*.

Table S2. Data collection and refinement statistics.

	WT_SeMet	WT_$P2_1$	WT_$P6_322$	WT_Glc	D394A-Nig2	D394A-Nig3	D394A-Nig4	D394A-Koj2
**Data collection**								
Beamline	AR-NW12A	AR-NW12A	AR-NW12A	AR-NW12A	BL5A	BL5A	AR-NW12A	AR-NW12A
Wavelength (Å)	0.9791	0.9791	1.0000	1.0000	1.0000	1.0000	1.0000	1.0000
Space group	$P6_322$	$P2_1$	$P6_322$	$P6_322$	$P6_322$	$P6_322$	$P6_322$	$P6_322$
Cell dimensions								
a, *b*, *c* (Å)	151.9, 151.9, 177.3	115.4, 207.9, 117.9	151.6, 151.6, 176.6	151.4, 151.4, 177.3	151.4, 151.4, 177.2	152.0, 152.2, 117.7	151.8, 151.8, 177.7	151.7, 151.7, 177.7
α, β, γ (º)	90, 90, 120	90, 103.6, 90	90, 90, 120	90, 90, 120	90, 90, 120	90, 90, 120	90, 90, 120	90, 90, 120
Resolution range (Å)	50–2.05 (2.16–2.05)	50–1.75 (1.84–1.75)	50–1.85 (1.95–1.85)	50–2.00 (2.11–2.00)	50–1.75 (1.84–1.75)	50–1.80 (1.90–1.80)	50–1.80 (1.90–1.80)	50–1.80 (1.90–1.80)
Measured reflections	3,040,423	3,635,745	2,006,600	3,211,165	2,381,021	2,192,135	2,190,359	2,192,732
Unique reflections	75,900	529,214	101,843	81,029	120,135	111,675	111,334	111,324
Completeness (%)	100 (100)[a]	98.0 (97.0)	100 (100)	100 (100)	100 (100)	100 (100)	100 (100)	100 (100)
Redundancy	40.1 (40.6)	6.9 (6.9)	19.7 (18.6)	39.6 (40.3)	19.8 (20.0)	19.6 (20.2)	19.7 (18.9)	19.7 (18.9)
Mean $I/\sigma(I)$	33.5 (5.6)	13.6 (1.5)	24.7 (3.0)	42.0 (4.7)	30.4 (2.9)	30.5 (2.6)	22.2 (3.3)	25.0 (2.8)
R_{merge}	0.123 (0.962)	0.092 (1.240)	0.091 (1.144)	0.091 (1.151)	0.067 (1.191)	0.072 (1.505)	0.095 (1.068)	0.090 (1.235)
$CC_{1/2}$ (7)	1.000 (0.950)	0.999 (0.642)	1.000 (0.882)	1.000 (0.947)	1.000 (0.869)	1.000 (0.841)	1.000 (0.882)	1.000 (0.848)
**Refinement statistics**								
R_{work}/R_{free}		0.185/0.216	0.161/0.184	0.181/0.208	0.166/0.190	0.176/0.194	0.167/0.189	0.170/0.191
RMSD[b]								
Bond length (Å)		0.010	0.010	0.012	0.011	0.010	0.010	0.010
Bond angles (º)		1.640	1.577	1.695	1.613	1.595	1.575	1.563
Noumber of atoms								
Protein		36,097	6,023	6,025	6,017	6,003	6,014	6,017
Ligand		107	27	43	62	53	65	55
Water		2879	577	351	552	480	638	580
Average *B* (Å^2)								
Protein		31.6	33.7	41.0	34.0	37.5	31.6	32.3
Ligands		30.3	54.5	45.3	43.2	37.9	42.9	40.8
Water		33.6	39.5	39.5	37.7	38.7	36.9	36.3
Ramachandran plot								
Favored (%)		95.38	95.25	94.92	96.46	96.97	96.30	96.15
Outliers (%)		0.02	0.14	0.14	0.00	0.00	0.00	0.00
Clash score		2.47	1.35	1.51	1.59	1.51	1.34	1.26
PDB codes		7WJ9	7WJA	7WJB	7WJC	7WJD	7WJE	7WJF

[a] The values for the highest resolution shells are given in parentheses.
[b] Root mean square deviation.

Table S3. Data collection, processing, and reconstruction statistics for cryo-EM.

(EMDB/PDB ID)	LlGH31_u1 (EMDB-32571/7WLG)
**Data collection and processing**	
Microscope	Talos Arctica
Voltage (kV)	200
Detector	Falcon 3EC
Magnification	120,000
Pixel size (Å)	0.88
Automation software	EPU
Total exposure (e–/Å^2)	50
Exposure rate (e–/Å^2 frames)	1.00
Number of frames	50
Defocus range (μm)	-0.8, -1.2, -1.6, -2.0
Number of collected micrograph	995 (491 + 504)
Number of particles for Class2D	267,294 (139,450 + 127,844)
Number of particles for Class3D	244,657 (127,990 + 116,667)
Number of particles for Refine3D	44,606
Symmetry imposed	D3
Map resolution (Å)	2.73
FSC threshold	0.143
Map resolution range (Å)	2.60-5.04
**Refinement**	
Initial model used (PDB code)	7WJA
Model composition	
Non-hydrogen atoms	35928
Protein residues	4368
Ligands	0
B factors (Å^2)	
Protein	53.8
Ligand	0
Map-model CC	
CC (mask)	0.87
CC (box)	0.78
CC (peaks)	0.74
CC (volume)	0.84
R.m.s. deviations	
Bond lengths (Å)	0.003
Bond angles (º)	0.495
Validation	
MolProbity score	1.55
Clash score	6.1
Rotamer outliers (%)	0.0
Ramachandran plot	
Favored (%)	96.7
Allowed (%)	3.4
Outliers (%)	0.0

Table S4. Oligonucleotide primers used in this study

Name	Sequence (5’ to 3’)[a]	Purpose
LlGH31u1_NdeI_F	CGGCAGCCATATGGTCAGTGAATTAGAAAGTAAA	Cloning of LlGH31_u1 into pET28a
LlGH31u1_NotI_R	CGAGTGCGGCCGCTTATTTTCCACTGTAAAGTAATTC	
CmGH31u1_NdeI_F	CGGCAGCCATATGCCATCATCCGCCTTCGTTGGTG	Cloning of CmGH31_u1 into pET28a
CmGH31u1_HindIII_R	GCCGCAAGCTTCTAGGATCGGCTGTCGGCTAGGAAG	
LlGH31u1_D394A_F	TCGGGAGCTTCAGTCATTTCATGGGCA	For construction of expression plasmid for inactive mutant D394A
LlGH31u1_D394A_R	GACTGAAGCTCCCGAAAATCCTAAAGG	
LlGH31u1_Y99F_F	TCTGTTTTCCAAATCGCTGGTATTTTG	For construction of expression plasmid for LlGH31_u1_Y99F mutant
LlGH31u1_Y99F_R	GATTTGGAAAACAGAAAAGTTAAATTT	

[a] Restriction sites are underlined.

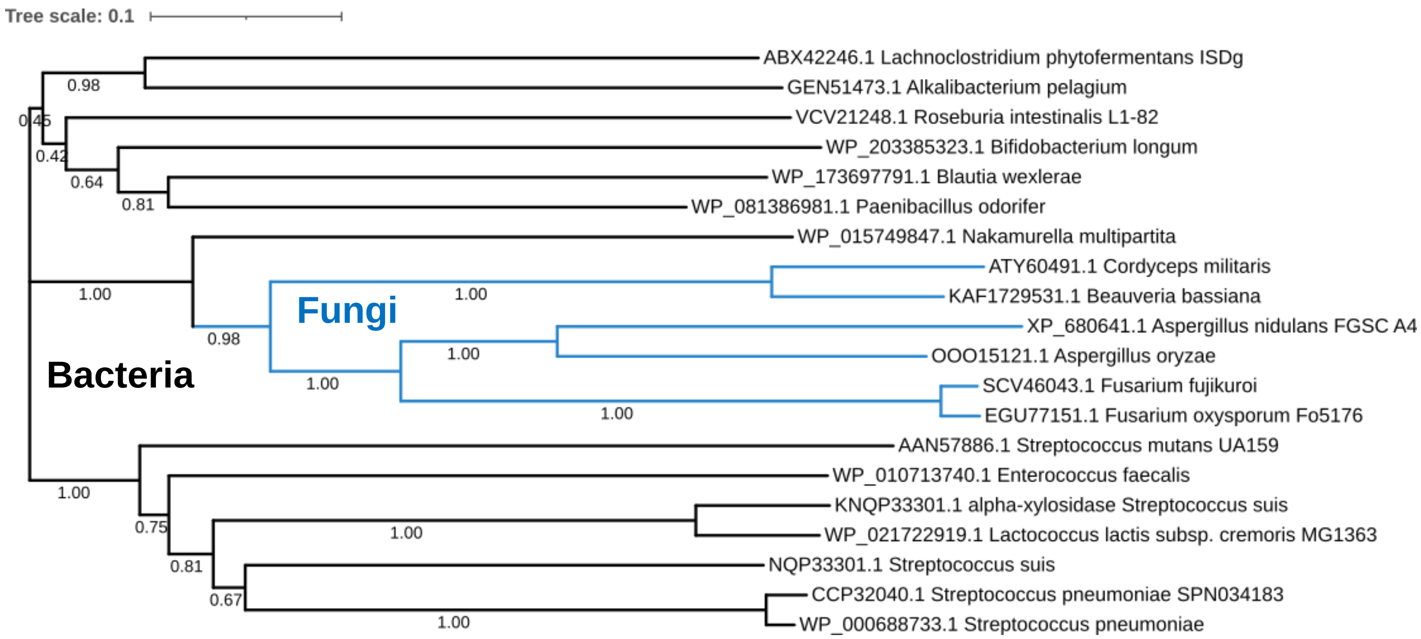

Figure S1. Phylogenetic tree of GH31_u1 proteins. GH31_u1 sequences were aligned using the MUSCLE algorithm, and the phylogenetic tree was built by the neighbor-joining method using MEGA X (9). Bootstrap values based on 1,000 replicates are shown. The tree was visualized using the iTOL v6 server (10).

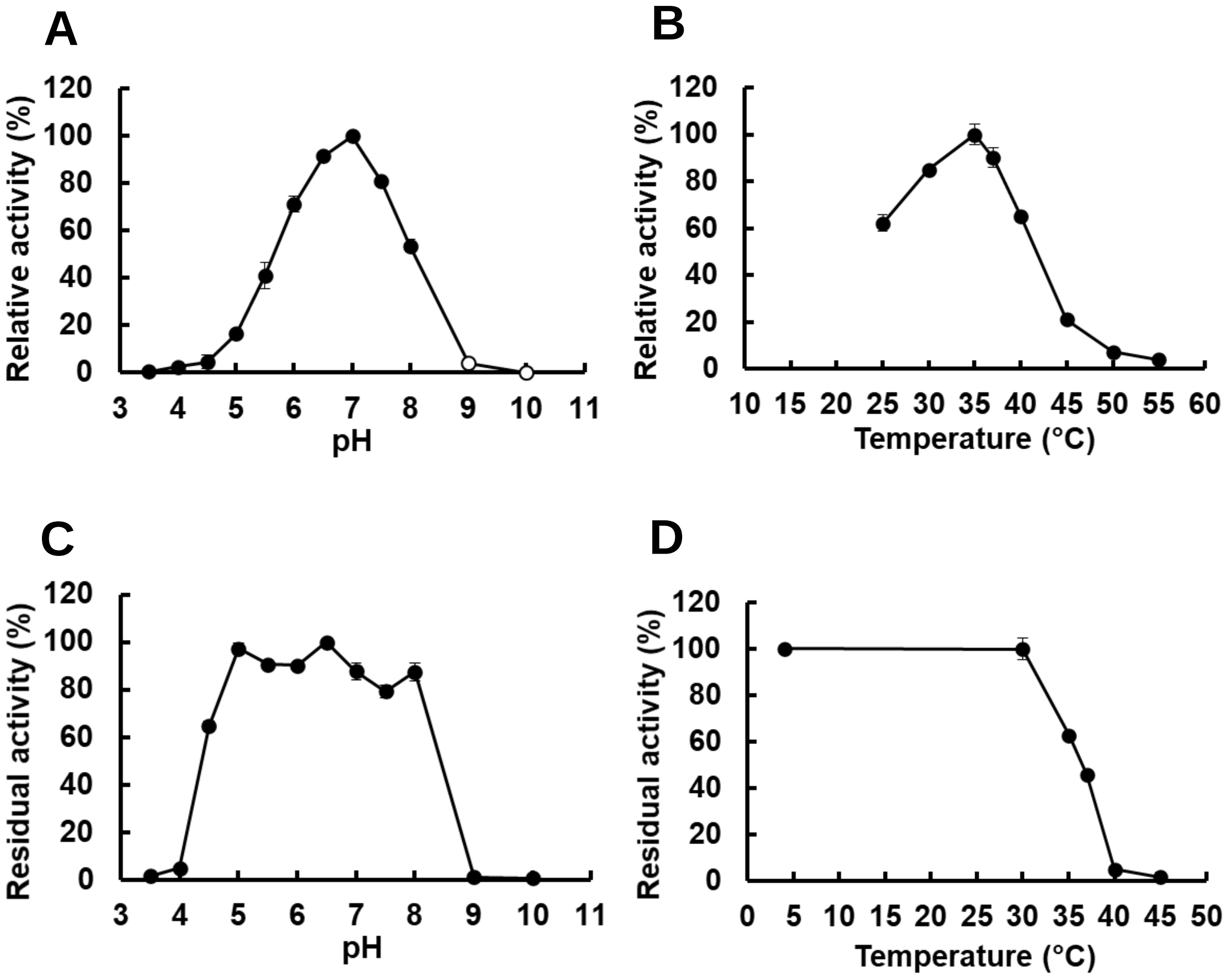

Figure S2. Effects of pH and temperature on the hydrolytic activity of LlGH31_u1. pH dependence (A), temperature dependence (B), pH stability (C), and thermostability (D) of LlGH31_u1 were determined using pNP-α-Glc as a substrate. pH dependence was measured in McIlvaine buffer at pH 3.5–8.0 or in glycine–HCl buffer at pH 9.0–10. Temperature dependence was evaluated in 50 mM sodium phosphate buffer (pH 7.0) at 25℃–55℃. pH stability was measured at 30℃ after incubating for 24 h in McIlvaine buffer at pH 3.5–8.0 or in glycine-HCl buffer at pH 9.0–10 and 4℃. Thermostability was measured at 30℃ after incubation in 50-mM sodium phosphate buffer (pH 7.0) at 4℃–45℃. All experiments were conducted in triplicate.

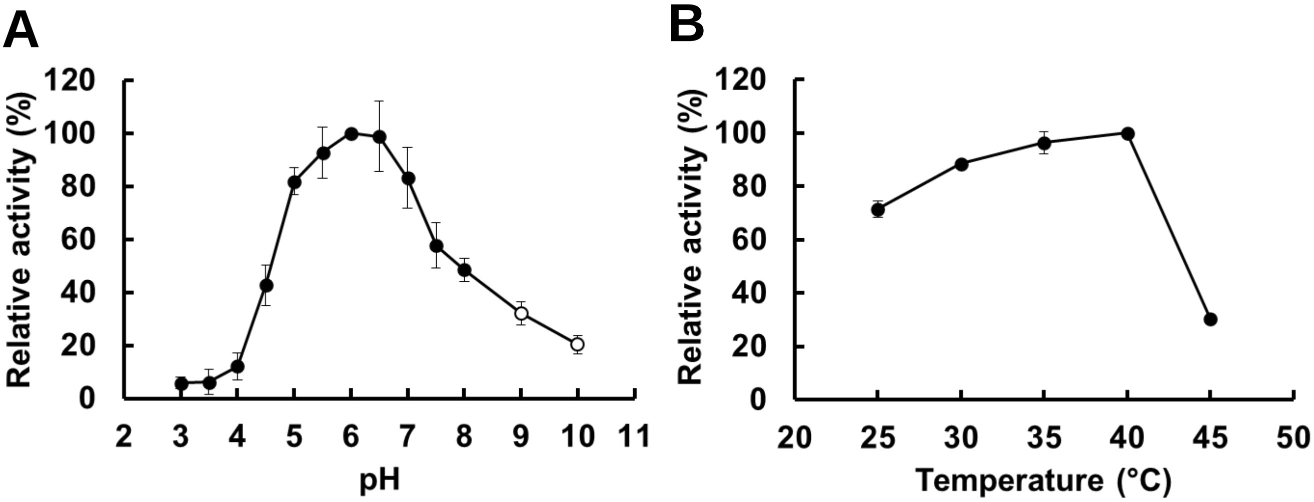

Figure S3. Effects of pH and temperature on the hydrolytic activity of CmGH31_u1. pH dependence (A) and temperature dependence (B) of CmGH31_u1 were determined using pNP-α-Glc as a substrate. pH dependence was measured in McIlvaine buffer at pH 3.5–8.0 or in glycine–HCl buffer at pH 9–10. Temperature dependence was examined in 50 mM sodium phosphate buffer (pH 7.0) at 25℃–55℃. All experiments were conducted in triplicate.

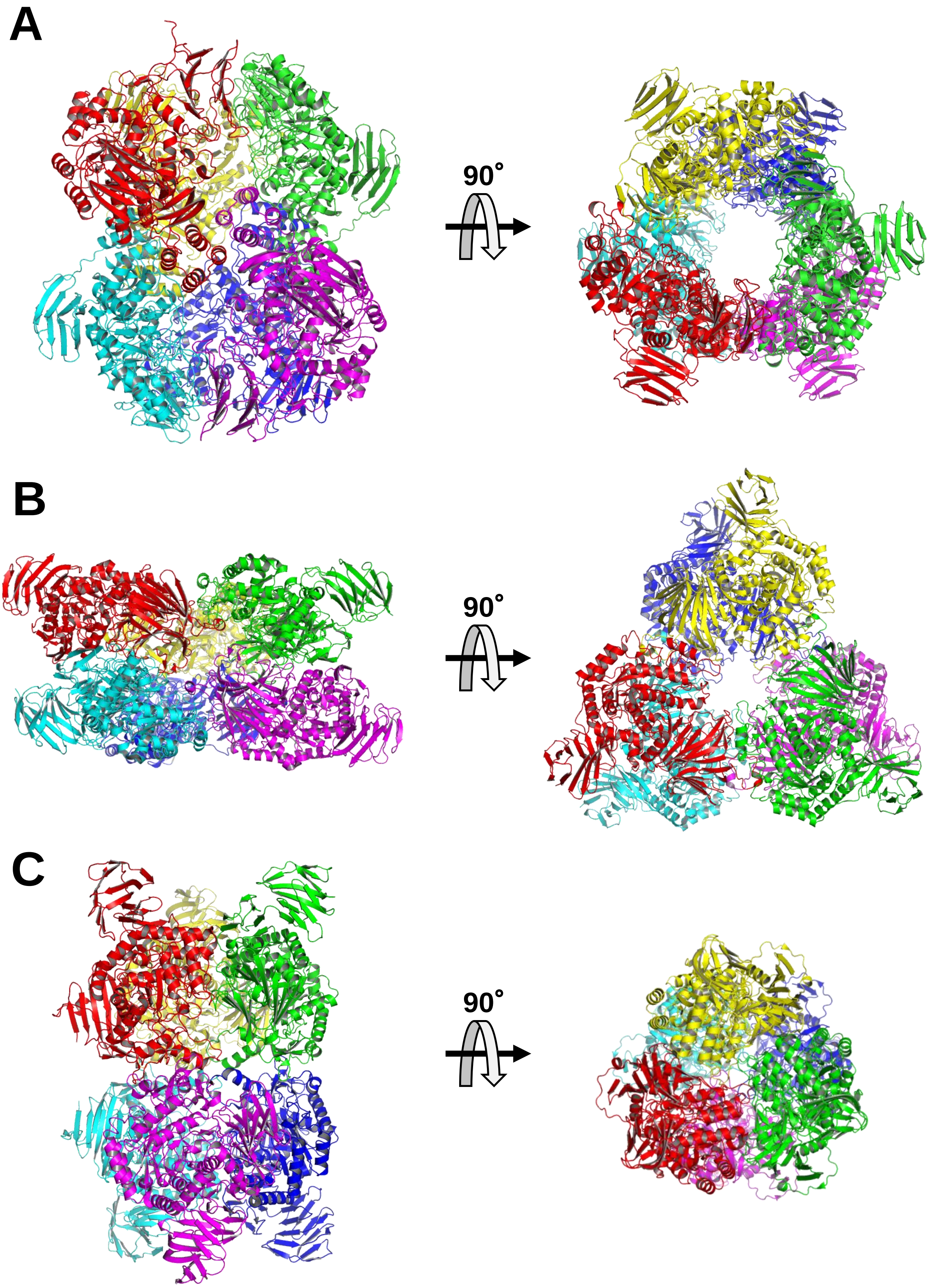

Figure S4. Comparison of the hexameric structures of GH31 enzymes. Ribbon diagrams of the hexameric structures of LlGH31_u1 (A), YicI (PDB 1XSJ) (B), and MalA (PDB 2G3M) (C) are shown in ribbon models.

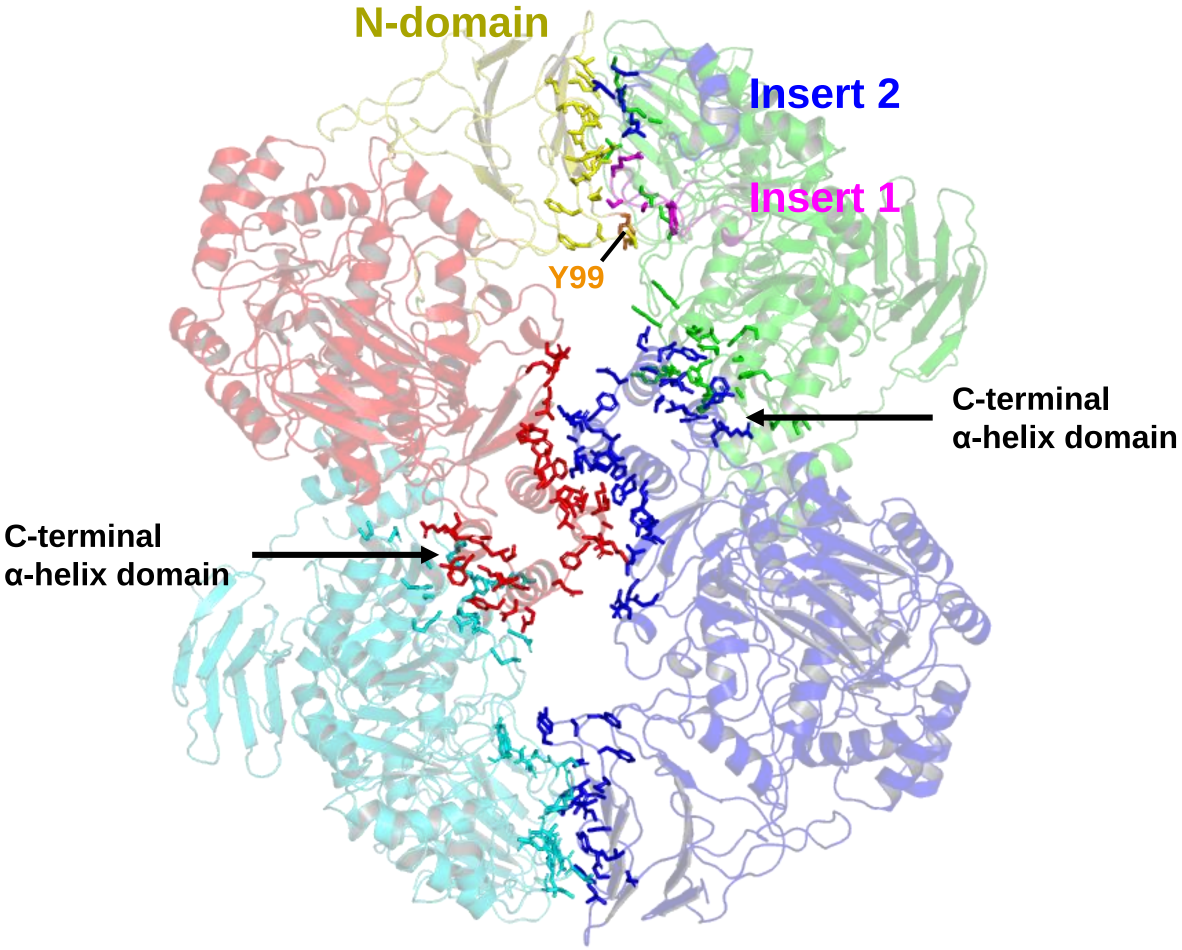

Figure S5. Amino acid residues on the interface of LlGH31_u1 hexamer. Four protomers (chains A, C, D, and E) of the cryo-EM structure are shown in ribbon models. Residues involved in hexamer formation are shown as stick models. N-domain of Chain A is colored yellow, Tyr99 is orange, and the other part of Chain A is red. Insert 1, and insert 2 of chain C are shown in magenta and blue, respectively, and the other part is green. Chain D and E are colored cyan and blue, respectively.

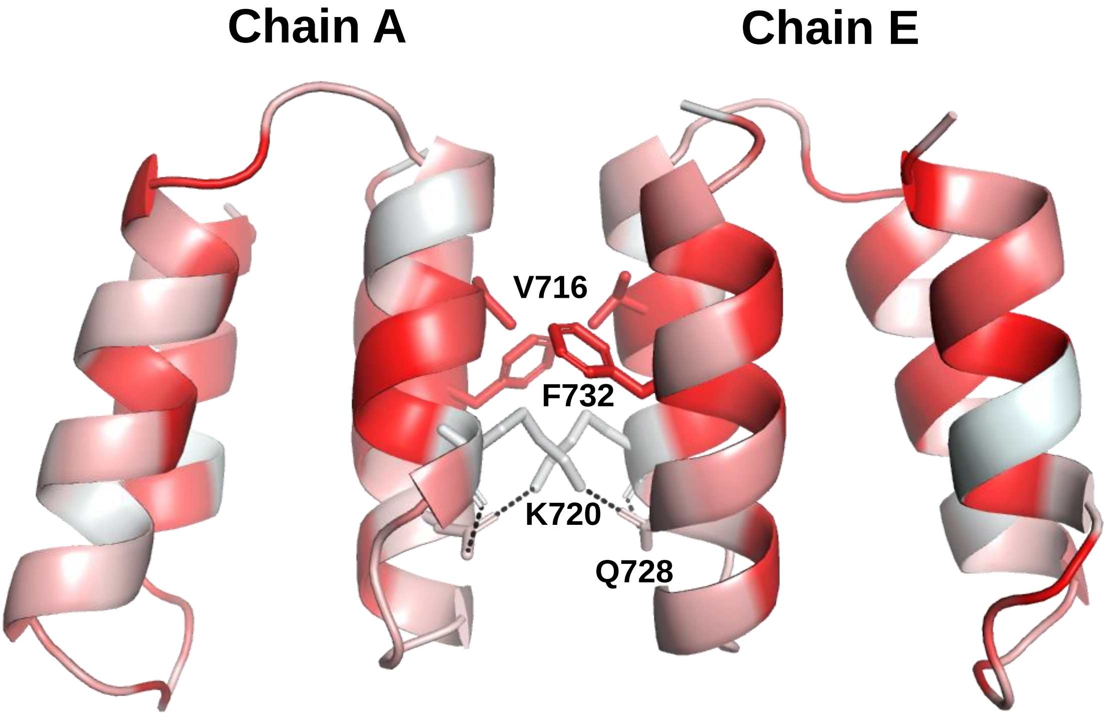

Figure S6. Interface residues of C-terminal α-helix domain. Structure of the C-terminal α-helix domain determined via cryo-EM is shown in ribbon models, and the side chains of amino acid residues that form hydrogen bonds (Lys720 and Gln728) and hydrophobic interaction (Val716 and Phe732) are shown in stick models. Dashed lines indicate hydrogen bonds. Residues are colored based on their hydrophobicity using the PyMOL color h script (https://pymolwiki.org/index.php/Color_h).

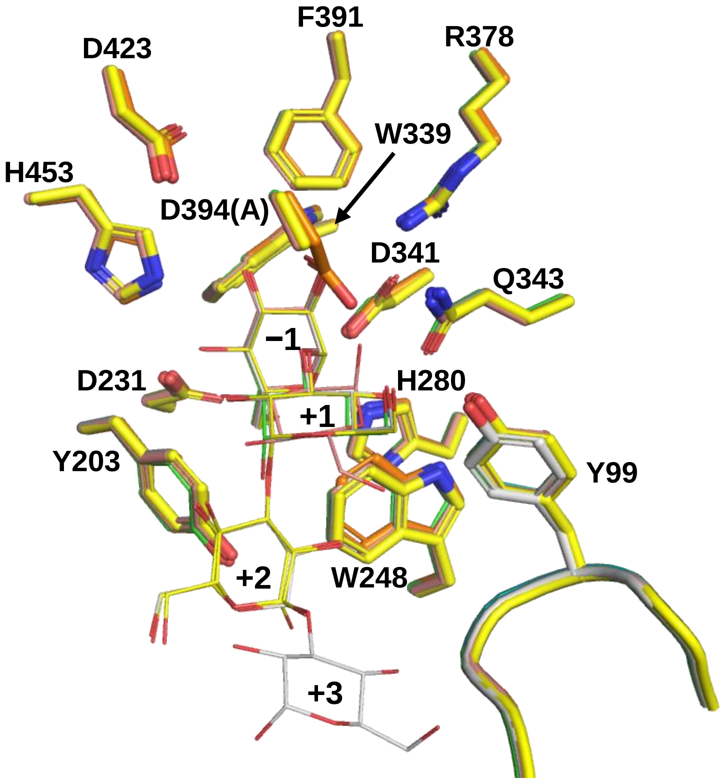

Figure S7. Superimposition of ligand-free and ligand-complex structures. Crystal structures of WT_$P6_322$ (orange), WT_Glc (cyan), D394A_Nig2 (green), D394A_Nig3 (yellow), D394A_Nig4 (white), and D394A_Koj2 (pink) are superimposed. Residues involved in substrate recognition are shown in stick models.

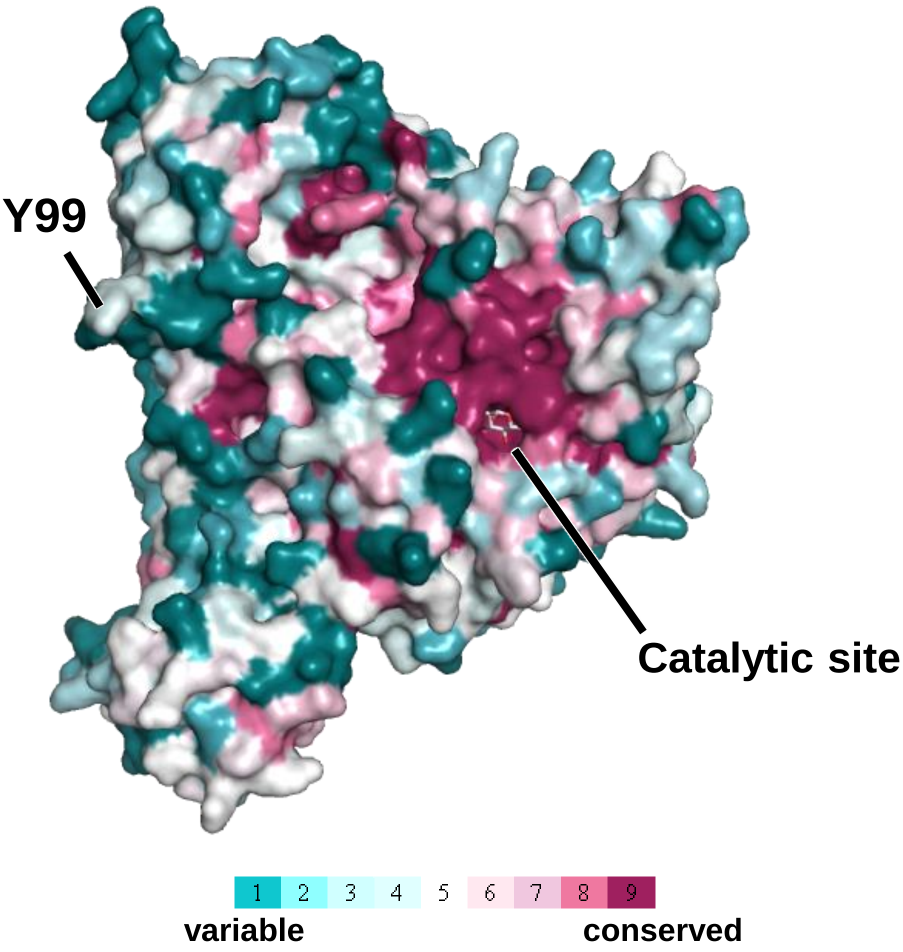

Figure S8. Residues conserved among GH31_u1 homologs. Conservation analysis was conducted using the ConSurf server (https://consurf.tau.ac.il/) (11–13). GH31_u1 homologs were searched via the HMMER method with an E-value cutoff of 0.0001 and sequence identity range of 35–95%. In total, 150 sequences were used to perform multiple sequence alignment and confirmed that they belong to the GH31_u1 cluster using the CUPP program (14).

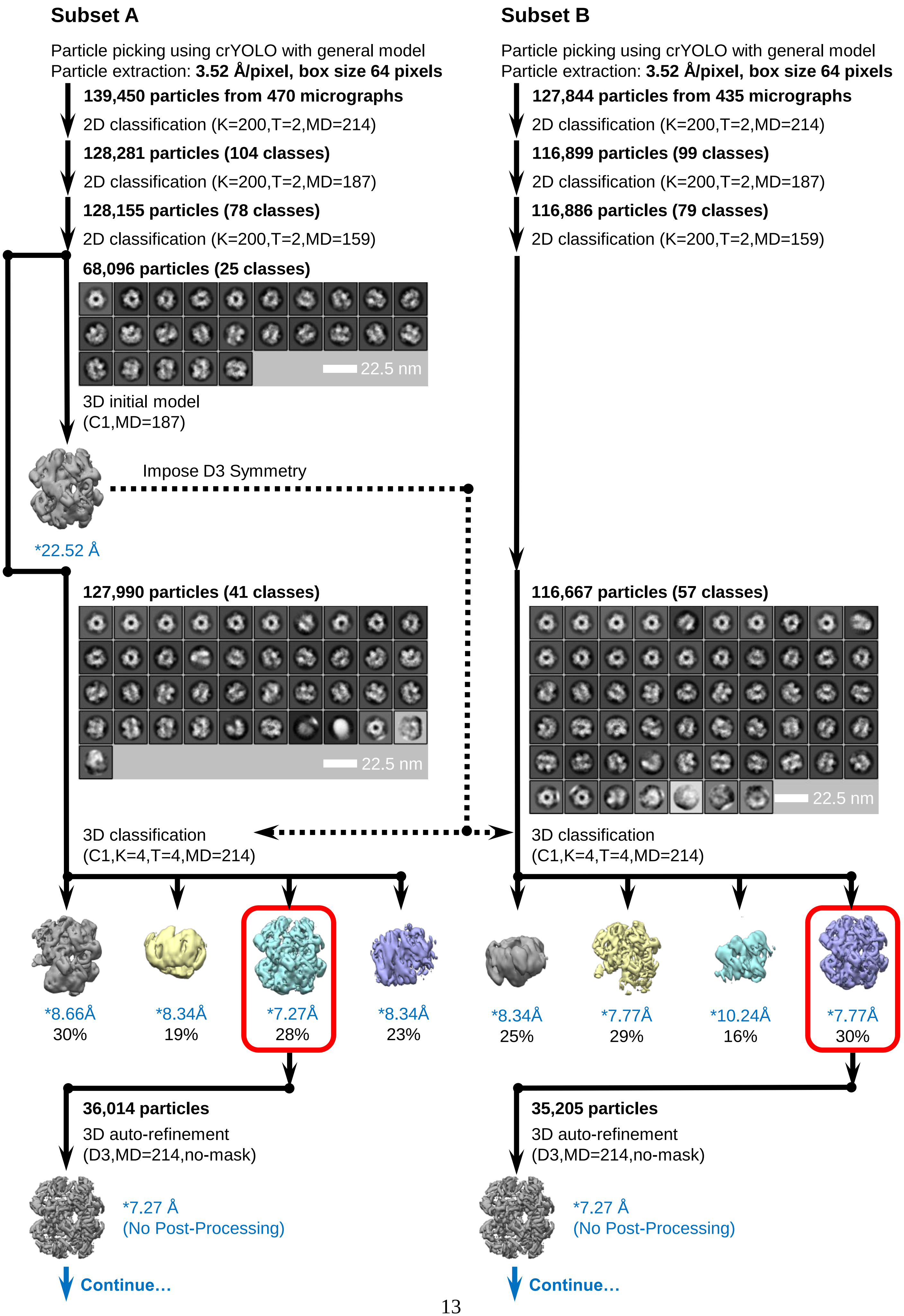
Subset A
Particle picking using crYOLO with general model
Particle extraction: 3.52 Å/pixel, box size 64 pixels
139,450 particles from 470 micrographs
2D classification (K=200,T=2,MD=214)
128,281 particles (104 classes)
2D classification (K=200,T=2,MD=187)
128,155 particles (78 classes)
2D classification (K=200,T=2,MD=159)
68,096 particles (25 classes)
22.5 nm
3D initial model
(C1,MD=187)
Impose D3 Symmetry
*22.52 Å
127,990 particles (41 classes)
22.5 nm
3D classification
(C1,K=4,T=4,MD=214)
*8.66Å
30%
*8.34Å
19%
*7.27Å
28%
*8.34Å
23%
36,014 particles
3D auto-refinement
(D3,MD=214,no-mask)
*7.27 Å
(No Post-Processing)
Continue…
Subset B
Particle picking using crYOLO with general model
Particle extraction: 3.52 Å/pixel, box size 64 pixels
127,844 particles from 435 micrographs
2D classification (K=200,T=2,MD=214)
116,899 particles (99 classes)
2D classification (K=200,T=2,MD=187)
116,886 particles (79 classes)
2D classification (K=200,T=2,MD=159)
116,667 particles (57 classes)
22.5 nm
3D classification
(C1,K=4,T=4,MD=214)
*8.34Å
25%
*7.77Å
29%
*10.24Å
16%
*7.77Å
30%
35,205 particles
3D auto-refinement
(D3,MD=214,no-mask)
*7.27 Å
(No Post-Processing)
Continue…

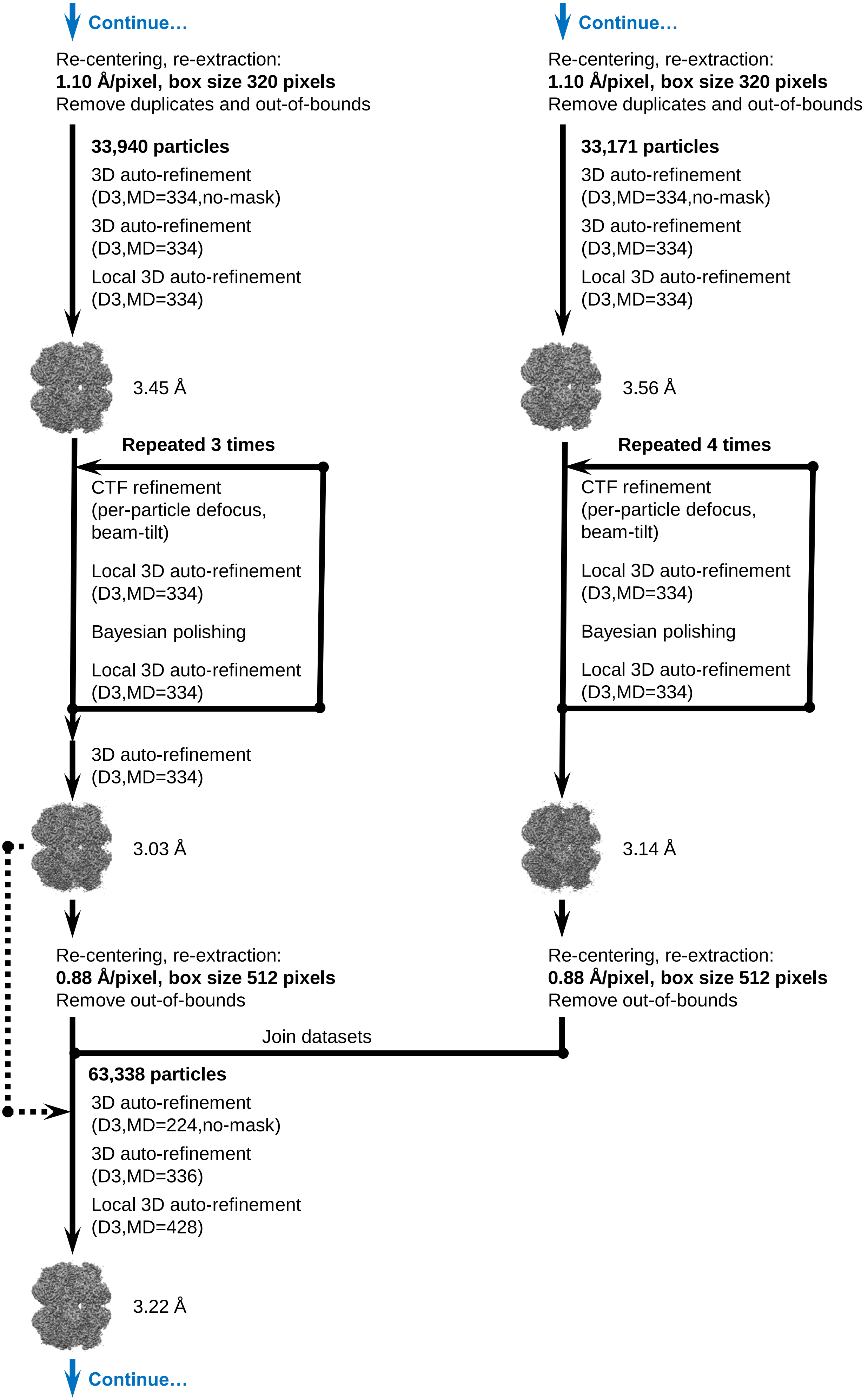
Continue…
Re-centering, re-extraction:
1.10 Å/pixel, box size 320 pixels
Remove duplicates and out-of-bounds
33,940 particles
3D auto-refinement
(D3,MD=334,no-mask)
3D auto-refinement
(D3,MD=334)
Local 3D auto-refinement
(D3,MD=334)
3.45 Å
Repeated 3 times
CTF refinement
(per-particle defocus,
beam-tilt)
Local 3D auto-refinement
(D3,MD=334)
Bayesian polishing
Local 3D auto-refinement
(D3,MD=334)
3D auto-refinement
(D3,MD=334)
3.03 Å
Re-centering, re-extraction:
0.88 Å/pixel, box size 512 pixels
Remove out-of-bounds
Continue…
Re-centering, re-extraction:
1.10 Å/pixel, box size 320 pixels
Remove duplicates and out-of-bounds
33,171 particles
3D auto-refinement
(D3,MD=334,no-mask)
3D auto-refinement
(D3,MD=334)
Local 3D auto-refinement
(D3,MD=334)
3.56 Å
Repeated 4 times
CTF refinement
(per-particle defocus,
beam-tilt)
Local 3D auto-refinement
(D3,MD=334)
Bayesian polishing
Local 3D auto-refinement
(D3,MD=334)
3.14 Å
Re-centering, re-extraction:
0.88 Å/pixel, box size 512 pixels
Remove out-of-bounds
Join datasets
63,338 particles
3D auto-refinement
(D3,MD=224,no-mask)
3D auto-refinement
(D3,MD=336)
Local 3D auto-refinement
(D3,MD=428)
3.22 Å
Continue…

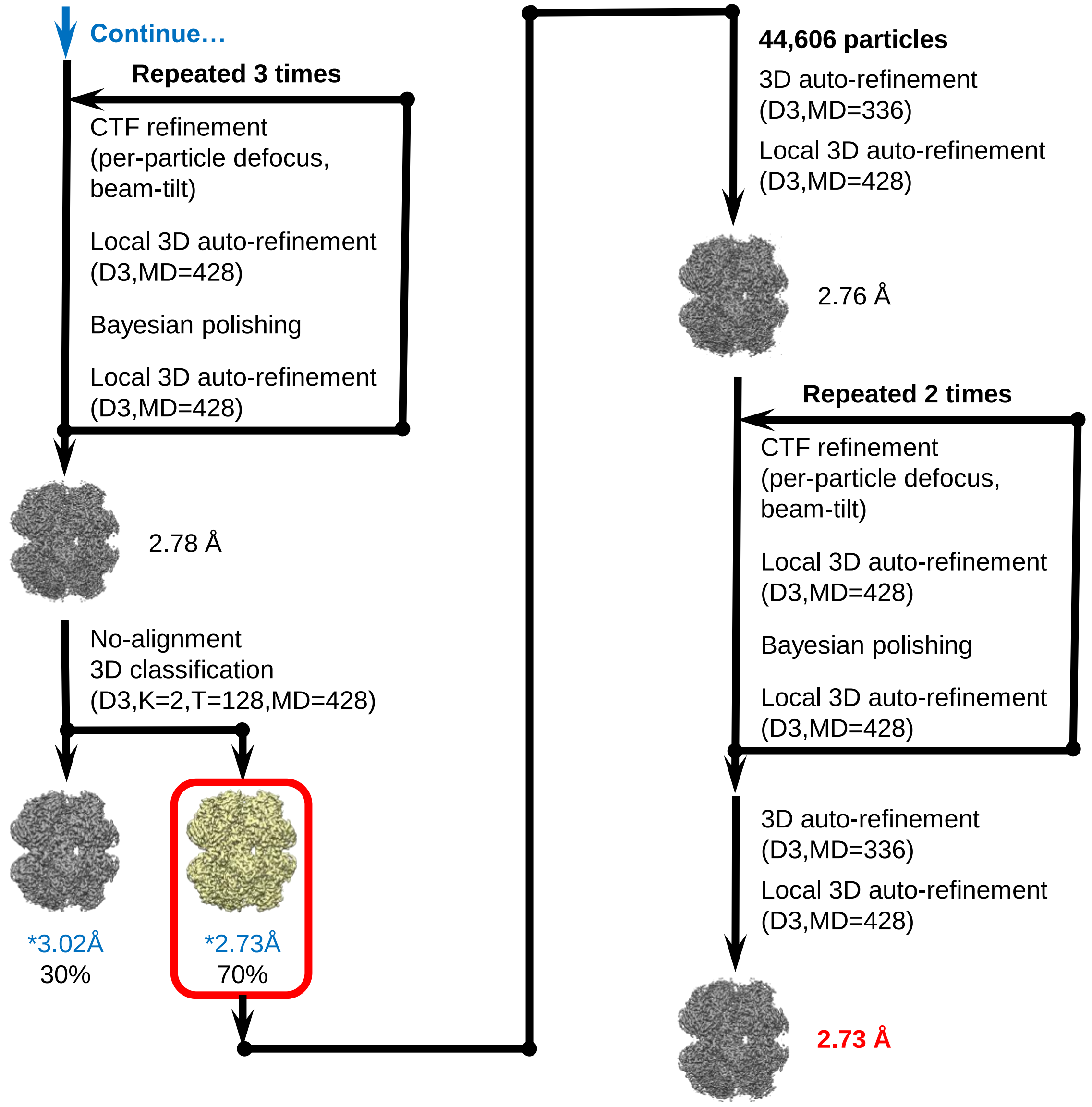

Figure S9. Cryo-EM data processing workflow. A detailed description is given in the Supplementary Methods section. Mask diameter in Å is abbreviated MD.

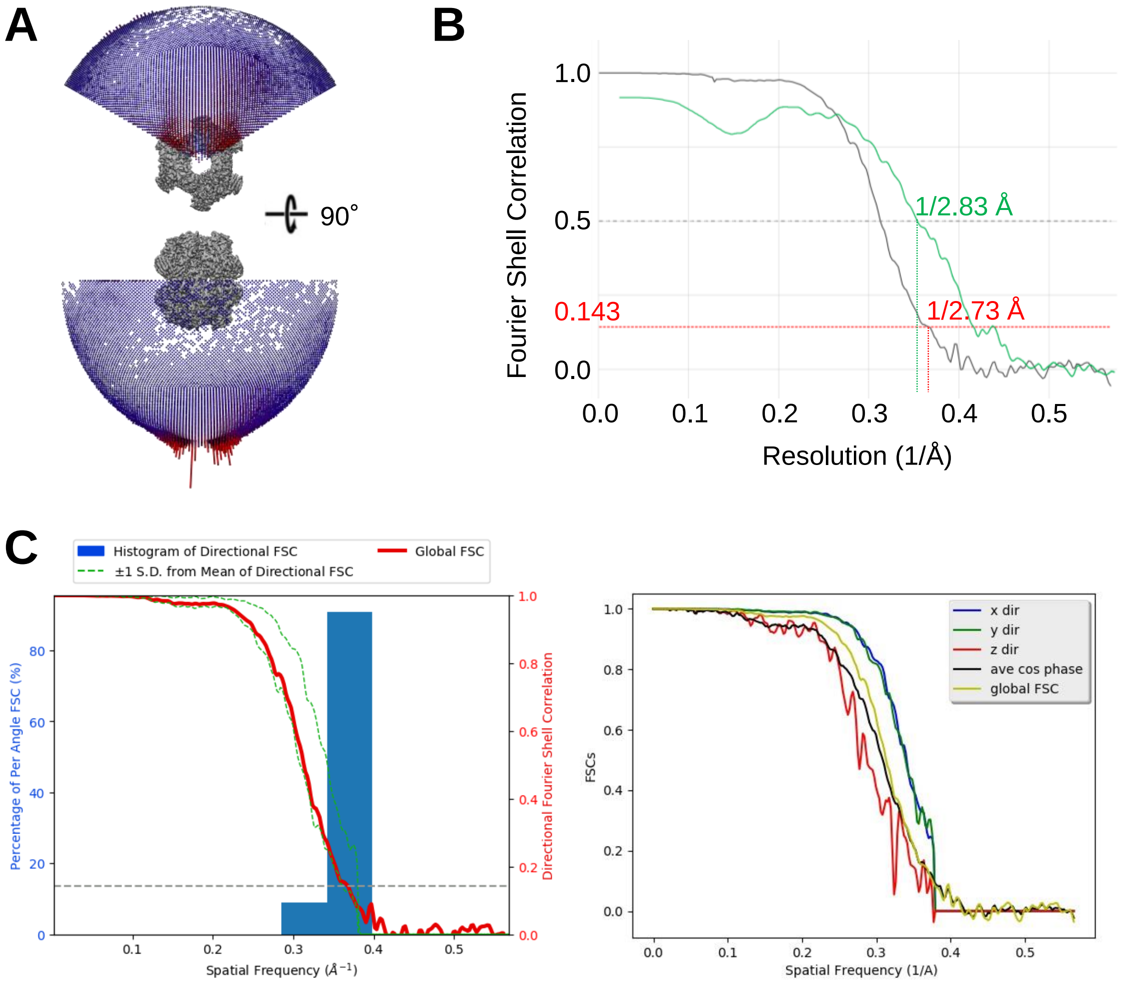

Figure S10. Validation of cryo-EM single-particle analysis. Orientation distributions (A), half-maps (black) and map-to-model FSC (green) curves (B), and the 3D FSC of the cryo-EM structure (15) (C). Left in (C): histogram of 100 directional resolutions evenly sampled over the 3D FSC (blue bars) and spread of the directional resolution of 3D FSC (the area encompassed by green dotted lines indicates ±1 standard deviation from the mean of the directional resolution (red line)). Right in (C): directional 3D FSC plots. The sphericity is 0.968 out of 1.

References

1. Zivanov, J., Nakane, T., and Scheres, S. H. W. (2020) Estimation of high-order aberrations and anisotropic magnification from cryo-EM data sets in RELION-3.1. *IUCrJ* **7**, 253-267
2. Zivanov, J., Nakane, T., and Scheres, S. H. W. (2019) A Bayesian approach to beam-induced motion correction in cryo-EM single-particle analysis. *IUCrJ* **6**, 5-17
3. Kang, M. S., Okuyama, M., Mori, H., and Kimura, A. (2009) The first α-1,3-glucosidase from bacterial origin belonging to glycoside hydrolase family 31. *Biochimie* **91**, 1434-1442
4. Okuyama, M., Miyamoto, M., Matsuo, I., Iwamoto, S., Serizawa, R., Tanuma, M., Ma, M., Klahan, P., Kumagai, Y., Tagami, T., and Kimura, A. (2017) Substrate recognition of the catalytic α-subunit of glucosidase II from *Schizosaccharomyces pombe*. *Biosci Biotechnol Biochem* **81**, 1503-1511
5. Kita, A., Matsui, H., Somoto, A., Kimura, A., Takata, M., and Chiba, S. (1991) Substrate Specificity and Subsite Affinities of Crystalline α-Glucosidase from *Aspergillus niger*. *Agric Biol Chem* **55**, 2327-2335
6. Matsui, H., Sasaki, M., Takemasa, E., Kaneta, T., and Chiba, S. (1984) Kinetic studies on the substrate specificity and active site of rabbit muscle acid α-glucosidase. *J Biochem* **96**, 993-1004
7. Lee, B. H., Rose, D. R., Lin, A. H., Quezada-Calvillo, R., Nichols, B. L., and Hamaker, B. R. (2016) Contribution of the individual small intestinal α-glucosidases to digestion of unusual α-linked glycemic disaccharides. *J Agric Food Chem* **64**, 6487-6494
8. Nihira, T., Nakai, H., Chiku, K., and Kitaoka, M. (2012) Discovery of nigerose phosphorylase from *Clostridium phytofermentans*. *Appl Microbiol Biotechnol* **93**, 1513-1522
9. Kumar, S., Stecher, G., Li, M., Knyaz, C., and Tamura, K. (2018) MEGA X: molecular evolutionary genetics analysis across computing platforms. *Mol Biol Evol* **35**, 1547-1549
10. Letunic, I., and Bork, P. (2016) Interactive tree of life (iTOL) v3: an online tool for the display and annotation of phylogenetic and other trees. *Nucleic Acids Res* **44**, W242-245
11. Landau, M., Mayrose, I., Rosenberg, Y., Glaser, F., Martz, E., Pupko, T., and Ben-Tal, N. (2005) ConSurf 2005: the projection of evolutionary conservation scores of residues on protein structures. *Nucleic Acids Res* **33**, W299-302
12. Celniker, G., Nimrod, G., Ashkenazy, H., Glaser, F., Martz, E., Mayrose, I., Pupko, T., and Ben-Tal, N. (2013) ConSurf: using evolutionary data to raise testable hypotheses about protein function. *Isr J Chem* **53**, 199-206
13. Ashkenazy, H., Erez E., Martz E., Pupko T., and Ben-Tal N. (2010) ConSurf 2010: calculating evolutionary conservation in sequence and structure of proteins and nucleic acids. *Nucleic Acids Res* **38**, W529-533
14. Barrett, K., and Lange, L. (2019) Peptide-based functional annotation of carbohydrate-active enzymes by conserved unique peptide patterns (CUPP). *Biotechnol Biofuels* **12**, 102
15. Tan, Y. Z., Baldwin, P. R., Davis, J. H., Williamson, J. R., Potter, C. S., Carragher, B., and Lyumkis, D. (2017) Addressing preferred specimen orientation in single-particle cryo-EM through tilting. *Nat Methods* **14**, 793-796
